# Supplementary material for: Maternal diet and gut microbiome composition modulate early‐life immune development
Source: EMBO Mol Med. 2023 Jun 6;15(8):e17241. doi: 10.15252/emmm.202217241 (PMC10405054; doi:10.15252/emmm.202217241)
Supplement: Supplementary file 1 — Expanded View Figures PDF [file EMMM-15-e17241-s011.pdf]

Expanded View Figures

Figure EV1. Colonic transcriptome changes among 14SM FF, 14SM FR, and 13SM FR pups at age 15 days.

A PCA plot of the top 500 most variable genes among samples analyzed by RNA sequencing (*n* = 4 mice/group).
B Volcano plot showing colonic transcripts that were significantly enriched in 14SM FR or 13SM FR after correction for multiple comparisons using DESeq2 (*n* = 3–4 mice/group).
C mRNA expression of *Il12p40* and *Il23*, targeted transcripts of interest (*n* = 3–20 mice/group).
D mRNA expression of *Ifit1b1*, transcript identified by RNA sequencing as elevated in 13SM FR (*n* = 3–8 mice/group).
E mRNA expression of transcripts belonging to top 5 GO gene sets elevated in 14SM FR (*Nlrbp1* and *Ppp3r2*) or 14SM FF (*Igtp*, *Tgtp2*, *Gbp2*, *Nox1*, *Hspb2*, *Fos*, *Clca3a2*, *Itga5*, and *F13a1*). Colonic transcription levels were normalized by *Hprt*, which was the most stable among the three housekeeping genes tested (*Hprt*, *Hsp90*, *Gapdh*) (*n* = 3–8 mice/group).

Data information: Longitudinal data of mean ± SEM is shown for 14SM FF, 14SM FR, and 13SM FR pups at age 10–20 days (circles) with statistical significance calculated using two-way ANOVA factored on group and time point compared with the 14SM FR control. To the right of each longitudinal plot, we also report colonic transcript levels for pups born to germ-free (GF) FF or FR dams at age 15 days (squares) with statistical significance based on a one-way ANOVA with false-discovery adjustment using the Benjamini–Hochberg method. Outliers removed using ROUT method with Q = 10%. Data are from two independent experiments and represent biological replicates.

© 2023 The Authors

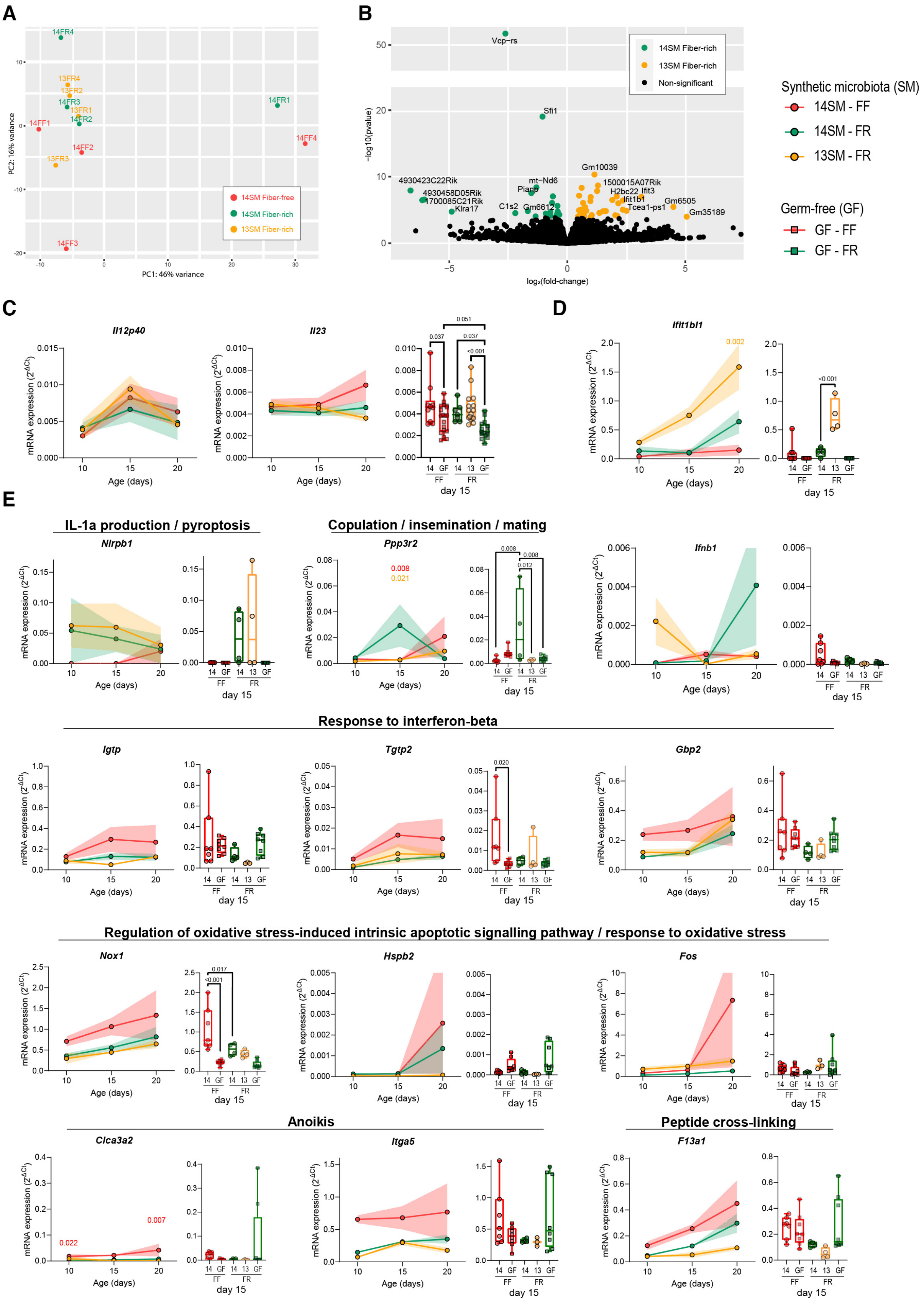

Figure EV1.

 © 2023 The Authors

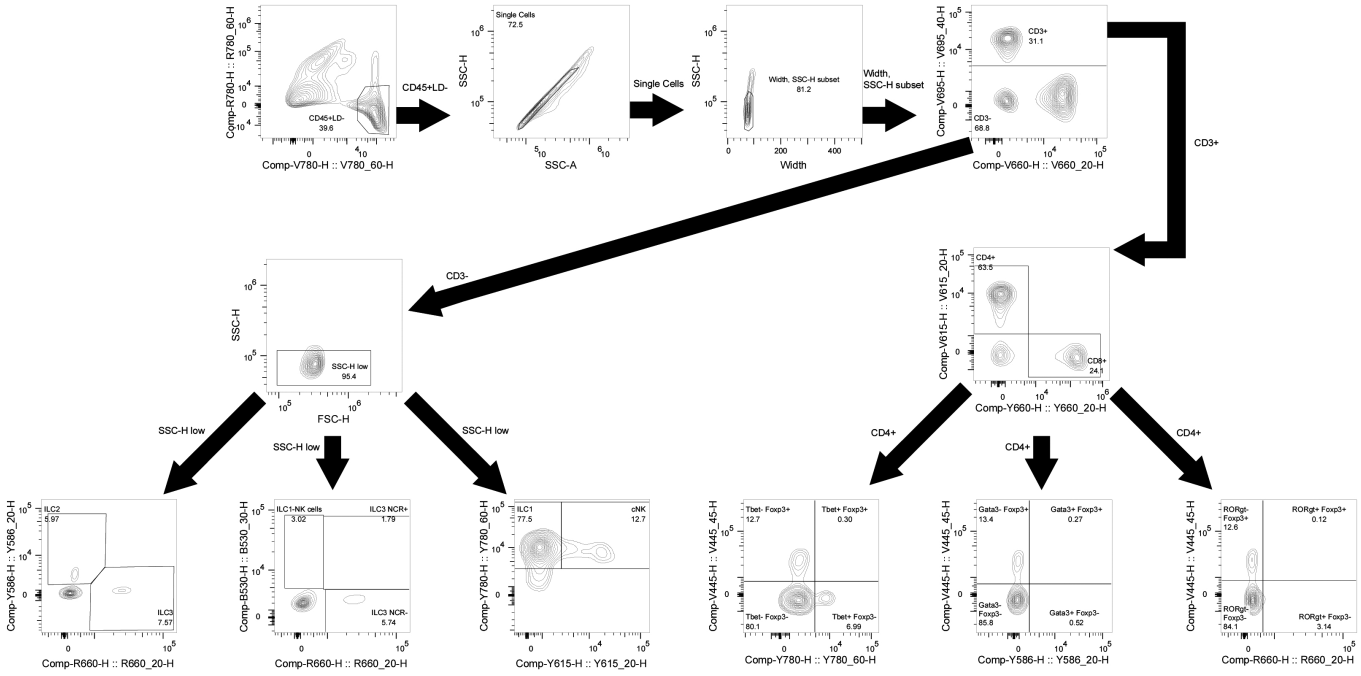

Figure EV2. Representative gating strategy for flow cytometry analysis.

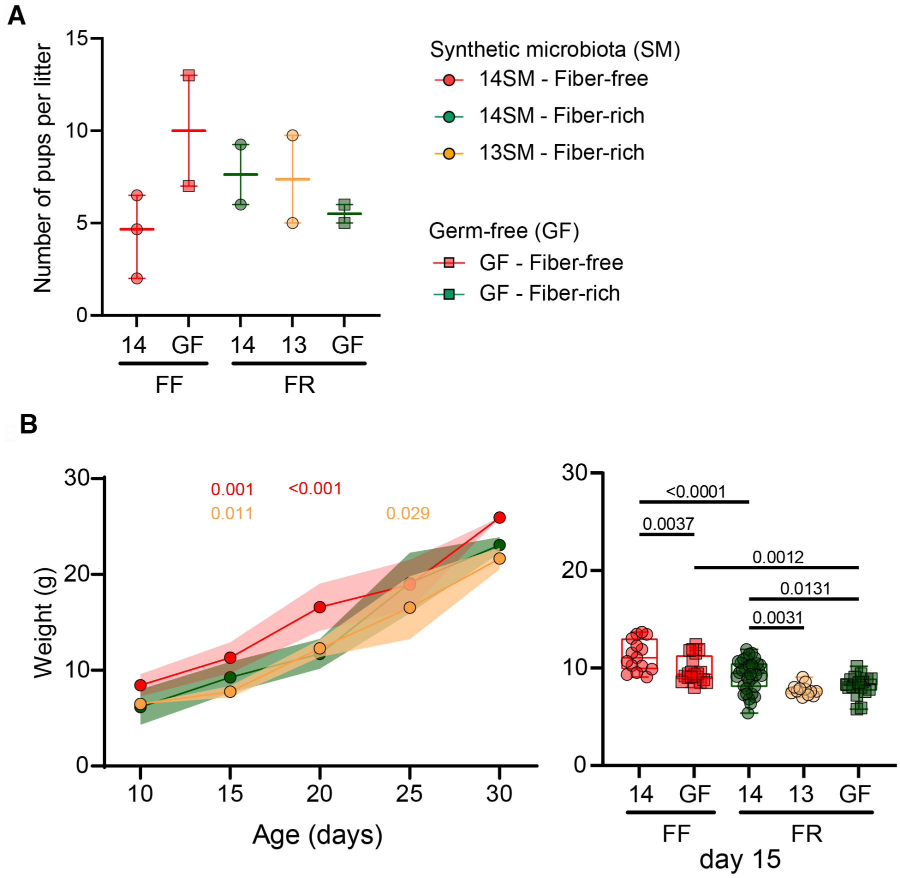

Figure EV3. Litter sizes and offspring weight gain among 14SM FF, 14SM FR, and 13SM FR groups.

A Number of pups born per litter in each group. (n = 2–3 l/group). No statistical significance for pairwise comparisons based on a one-way ANOVA with false-discovery adjustment using the Benjamini–Hochberg method.

B Pup weights at the specified intervals. (n = 2–17 mice/group). Longitudinal data of mean weight (g) ± SEM is shown for 14SM FF, 14SM FR, and 13SM FR pups at age 10–30 days (circles) with statistical significance calculated using two-way ANOVA factored on group and time point compared with the 14SM FR control. To the right, we also report weights of pups born to germ-free (GF) FF or FR dams at age 15 days (squares) with statistical significance based on a one-way ANOVA with false-discovery adjustment using the Benjamini and Hochberg method.

© 2023 The Authors

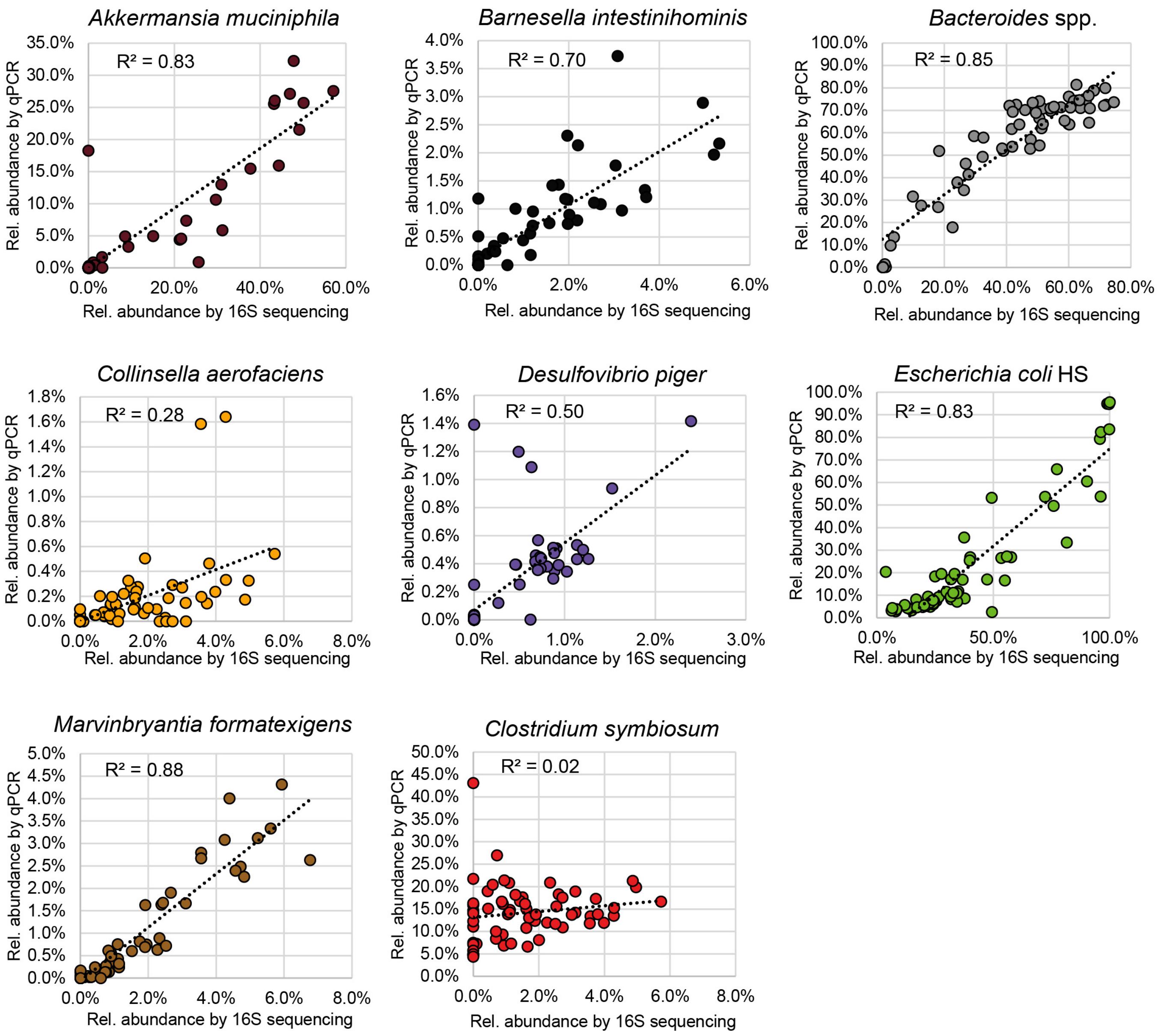

Figure EV4. Correlation of bacterial abundances determined by qPCR versus 16S rRNA gene sequencing.

Correlation coefficient (R^2) for the linear trendline fit to relative abundance by qPCR (y-axis) versus relative abundance by 16S rRNA gene sequencing (x-axis). Plots shown for all bacteria that were present at a detectable level in the pups of dams colonized with 13SM or 14SM.

© 2023 The Authors
