# Supplementary material for: Maternal diet and gut microbiome composition modulate early‐life immune development
Source: EMBO Mol Med. 2023 Jun 6;15(8):e17241. doi: 10.15252/emmm.202217241 (PMC10405054; doi:10.15252/emmm.202217241)
Supplement: Supplementary file 14 [file EMMM-15-e17241-s013.pdf]

EMBO Molecular Medicine

Maternal diet and gut microbiome composition modulate early life immune development

Mahesh Desai, Erica Grant, Marie Boudaud, Arnaud Muller, and Andrew Macpherson
DOI: 10.15252/emmm.202217241

Corresponding author: Mahesh Desai (mahesh.desai@lih.lu)

Review Timeline:

Submission Date:	29th Nov 22
Editorial Decision:	10th Jan 23
Revision Received:	24th Apr 23
Editorial Decision:	10th May 23
Revision Received:	15th May 23
Accepted:	16th May 23

Editor: Zeljko Durdevic

Transaction Report:

(Note: With the exception of the correction of typographical or spelling errors that could be a source of ambiguity, letters and reports are not edited. Depending on transfer agreements, referee reports obtained elsewhere may or may not be included in this compilation. Referee reports are anonymous unless the Referee chooses to sign their reports.)

10th Jan 2023

Dear Prof. Desai,

Thank you for the submission of your manuscript to EMBO Molecular Medicine and please accept my apologies for the delay in getting back to you due to the holiday season. We have now received feedback from the three reviewers who agreed to evaluate your manuscript. As you will see from the reports below, all three referees recognize potential interest of the study, but also raise serious and partially overlapping concerns particularly regarding the experimental setup as dams and pups were kept on a same diet.

Further consideration of a revision that addresses reviewers' concerns in full will entail a second round of review. EMBO Molecular Medicine encourages a single round of revision only and therefore, acceptance or rejection of the manuscript will depend on the completeness of your responses included in the next, final version of the manuscript. For this reason, and to save you from any frustrations in the end, I would strongly advise against returning an incomplete revision.

We would welcome the submission of a revised version within three months for further consideration. Please let us know if you require longer to complete the revision.

I look forward to receiving your revised manuscript.

Yours sincerely,

Zeljko Durdevic

Zeljko Durdevic
Editor
EMBO Molecular Medicine

*****

When submitting your revised manuscript, please carefully review the instructions that follow below. We perform an initial quality control of all revised manuscripts before re-review; failure to include requested items will delay the evaluation of your revision.

We require:

1) A .docx formatted version of the manuscript text (including legends for main figures, EV figures and tables). Please make sure that the changes are highlighted to be clearly visible.

2) Individual production quality figure files as .eps, .tif, .jpg (one file per figure). For guidance, download the 'Figure Guide PDF': (https://www.embopress.org/page/journal/17574684/authorguide#figureformat).

3) A .docx formatted letter INCLUDING the reviewers' reports and your detailed point-by-point responses to their comments. As part of the EMBO Press transparent editorial process, the point-by-point response is part of the Review Process File (RPF), which will be published alongside your paper.

4) A complete author checklist, which you can download from our author guidelines (https://www.embopress.org/page/journal/17574684/authorguide#submissionofrevisions). Please insert information in the checklist that is also reflected in the manuscript. The completed author checklist will also be part of the RPF.

5) Please note that all corresponding authors are required to supply an ORCID ID for their name upon submission of a revised manuscript.

6) It is mandatory to include a 'Data Availability' section after the Materials and Methods. Before submitting your revision, primary

datasets produced in this study need to be deposited in an appropriate public database, and the accession numbers and database listed under 'Data Availability'. Please remember to provide a reviewer password if the datasets are not yet public (see https://www.embopress.org/page/journal/17574684/authorguide#dataavailability).

In case you have no data that requires deposition in a public database, please state so in this section. Note that the Data Availability Section is restricted to new primary data that are part of this study.

7) For data quantification: please specify the name of the statistical test used to generate error bars and P values, the number (n) of independent experiments (specify technical or biological replicates) underlying each data point and the test used to calculate p-values in each figure legend. The figure legends should contain a basic description of n, P and the test applied. Graphs must include a description of the bars and the error bars (s.d., s.e.m.). See also 'Figure Legend' guidelines: https://www.embopress.org/page/journal/17574684/authorguide#figureformat

8) At EMBO Press we ask authors to provide source data for the main manuscript figures. Our source data coordinator will contact you to discuss which figure panels we would need source data for and will also provide you with helpful tips on how to upload and organize the files.

9) Our journal encourages inclusion of *data citations in the reference list* to directly cite datasets that were re-used and obtained from public databases. Data citations in the article text are distinct from normal bibliographical citations and should directly link to the database records from which the data can be accessed. In the main text, data citations are formatted as follows: "Data ref: Smith et al, 2001" or "Data ref: NCBI Sequence Read Archive PRJNA342805, 2017". In the Reference list, data citations must be labeled with "[DATASET]". A data reference must provide the database name, accession number/identifiers and a resolvable link to the landing page from which the data can be accessed at the end of the reference. Further instructions are available at .

10) We replaced Supplementary Information with Expanded View (EV) Figures and Tables that are collapsible/expandable online. A maximum of 5 EV Figures can be typeset. EV Figures should be cited as 'Figure EV1, Figure EV2" etc... in the text and their respective legends should be included in the main text after the legends of regular figures.

- For the figures that you do NOT wish to display as Expanded View figures, they should be bundled together with their legends in a single PDF file called *Appendix*, which should start with a short Table of Content. Appendix figures should be referred to in the main text as: "Appendix Figure S1, Appendix Figure S2" etc.

- Additional Tables/Datasets should be labeled and referred to as Table EV1, Dataset EV1, etc. Legends have to be provided in a separate tab in case of .xls files. Alternatively, the legend can be supplied as a separate text file (README) and zipped together with the Table/Dataset file.

See detailed instructions here:

.

11) The paper explained: EMBO Molecular Medicine articles are accompanied by a summary of the articles to emphasize the major findings in the paper and their medical implications for the non-specialist reader. Please provide a draft summary of your article highlighting

- the medical issue you are addressing,

- the results obtained and

- their clinical impact.

This may be edited to ensure that readers understand the significance and context of the research. Please refer to any of our published articles for an example.

12) For more information: There is space at the end of each article to list relevant web links for further consultation by our readers. Could you identify some relevant ones and provide such information as well? Some examples are patient associations, relevant databases, OMIM/proteins/genes links, author's websites, etc...

13) Author contributions: You will be asked to provide CRediT (Contributor Role Taxonomy) terms in the submission system. These replace a narrative author contribution section in the manuscript.

14) A Conflict of Interest statement should be provided in the main text.

15) Every published paper now includes a 'Synopsis' to further enhance discoverability. Synopses are displayed on the journal webpage and are freely accessible to all readers. They include a short stand first (maximum of 300 characters, including space) as well as 2-5 one-sentences bullet points that summarizes the paper. Please write the bullet points to summarize the key NEW findings. They should be designed to be complementary to the abstract - i.e. not repeat the same text. We encourage inclusion of key acronyms and quantitative information (maximum of 30 words / bullet point). Please use the passive voice. Please attach these in a separate file or send them by email, we will incorporate them accordingly.

Please also suggest a striking image or visual abstract to illustrate your article as a PNG file 550 px wide x 300-600 px high.

EMBO Molecular Medicine has a "scooping protection" policy, whereby similar findings that are published by others during review or revision are not a criterion for rejection. Should you decide to submit a revised version, I do ask that you get in touch after three months if you have not completed it, to update us on the status.

Please note: When submitting your revision you will be prompted to enter your funding and payment information. This will allow Wiley to send you a quote for the article processing charge (APC) in case of acceptance. This quote takes into account any reduction or fee waivers that you may be eligible for. Authors do not need to pay any fees before their manuscript is accepted and transferred to the publisher.

EMBO Press participates in many Publish and Read agreements that allow authors to publish Open Access with reduced/no publication charges. Check your eligibility: https://authorservices.wiley.com/author-resources/Journal-Authors/open-access/affiliation-policies-payments/index.html

***** Reviewer's comments *****

Referee #1 (Remarks for Author):

In this report, Grant and colleagues describe the evolution, in the intestine, of a consortium of 13 (no Akkermansia muciniphila, Am) or 14 bacteria (with Am) from age 10 days to age 30 days after birth. These mice are also given fiber-rich or fiber-free diets as an additional variable. RNA sequencing was performed on the colon of 15 days old mice, cytokines measured by qRT-PCR and different subsets of immune cells measured by flow cytometry.

This is an interesting study that yields many useful data. In particular, it analyzes the impact of Am not in isolation in monocolonized mice, but rather in the context of more complex microbiotas. But then, the data presented is complex, and it is difficult for the reader to connect all the dots. This is OK for cytokine measurements and cytometry of immune cells, but not OK for RNA sequencing data.

Furthermore, I would suggest that missing is a metabolomic analysis of the output of the different microbiotas in the context of different diets. This may allow identifying compounds that act on immune cells and thus, for the authors to generate a more mechanistic set of data to link diet, microbiota and immunity.

A few comments:

1. Figure 2D: values for Tnfa, Il17f and Il22 are rather low and highly variable. Are different experiments, cages of sexes pooled? If yes, this should be indicated on the plots (using different symbols for example) in order to appreciate the source of variability. The statistical method should also be adapted to account for potentially linked datasets.

2. Figure 3A: similar comment. In addition, several data group are bimodal in distribution (ILC3 in particular). What is the source of bimodal distribution here, as it might explain apparent differences between groups?

3. The data reported in Figure 2C is potentially interesting, but is left "hanging" in the study with no clear connections with the rest of the study (so not exploited). Some effort should be done to improve this issue.

Referee #2 (Remarks for Author):

Grant et al sought to study how fiber intake by the mother can imprint the immune system of the offspring, and how changes in the microbiota composition induced by the absence of fiber are involved in this process. They colonized mothers with a defined consortium of bacterial strains isolated from human feces and analyzed the impact of dietary fiber intake by mothers (and their pups) on the microbiota composition and on the development of the intestinal immune system at early life of the offspring. The

authors propose that pups of fiber deprived dams display a delayed colonization of Akkermansia muciniphila (AM), and that removal of the same species under fiber sufficient state results in a decreased RORgt-positive innate and adaptive immune cells. They concluded that A. muciniphila contributed to an early-life immune imprinting. Although the authors are addressing an important biological question - related to the interplay between diet, microbiota and intestinal immune responses - with potentially interesting implications, the data lacked coherence. Whereas the read-outs for microbiota composition are clear and conclusive, the read-outs utilized to characterize the immune responses, and whether (and how) A. muciniphila impact the "weaning reaction" are less clear, and its implications less conclusive. Therefore, I recommend revision and addition of new experiments to support the author's conclusions. See comments below.

General comments:
The authors chose a human consortium based on their previous paper DOI:https://doi.org/10.1016/j.cell.2016.10.043. However, it is not clear whether this consortium recapitulates the broad spectrum of immune responses observed in SPF mice. Are these bacterium species normally found in the microbiota of SPF mice? This is important considering the alteration of immune pathways related to microbiota and fiber intake the authors propose in their study. How do the immune parameters evaluated in this study compare to WT SPF mice? Can the authors clarify and discuss these points?

In the experimental setups used throughout the study, dams and pups are kept on a fiber-free or rich diet for the duration of the experiment. Even though the immune analyses are performed at early time points (presumably before the animals have started eating solid food), this could have a direct long-term impact on microbiota composition and intestinal immune system. To definitively conclude that the fiber intake by the mothers affect the immune system of the offspring, the pups born from mothers fed a fiber-free diet should also be given a fiber-rich diet at weaning age (d17-21 post-birth), and analyzed at d30 post-birth (same time point as the microbiota composition analysis). In other words, are the immune effects related to fiber intake by the mothers transient or long-term (see also specific comments for figure 3)?

Specific comments:
In Fig 1A, there appears to have a typo in the experimental scheme. It should be 13SM fiber-rich instead of fiber-full.

In Fig 1C, what are the implications for the changes in microbiota composition (besides AM) over time comparing fiber-free and fiber-rich diets? For example, what does the change in Dp or Bc mean?

In Fig S1, is the variance on PC1 due to batch effect (14FR1 and 14FF4 compared to rest)? Also, this PCA plot shows that groups are comparable (no major differences between the groups), could the authors comment on this?

The model in Fig 4 shows that under control condition (14SM, Fiber rich diet), AM expands and shrinks over the weaning period. However, the data in Figure 1B (middle panel) and 1C (AM panel) does not show clearly whether AM is significantly different across the different ages of the pups. It looks like D20 and D30 are very similar in fiber-free diet group whereas D15 and D30 are very similar in the fiber-rich diet. This is particularly important because at D20 the pups have started eating solid food. Can the authors explain this?

In Fig 2, how do the authors reconcile the differences in mRNA expression of weaning reaction cytokines (Ifng and Tnfa) in the colon of mice described in this study compared to the study of Al Nabahani et al, 2019 in which the authors show increase mRNA expression of ifng and Tnfa in the ileum of SPF mice at D21 but not at D15 post-birth? Can the authors explain why D20 in the present study is similar to D10 post-birth but elevated at D15 post-birth? In addition, how the authors explain the role of diet on the cytokine mRNA expression in germ-free mice since they are not exposed to microbiota during the weaning period and therefore do not experience the so-called "weaning reaction"? Were the outliers removed for statistical analysis but included in the graph? Since mRNA expression by qPCR does not follow a linear distribution, were the data log-transformed prior to stat analysis?

In Fig 2A, it is surprising that the number of enriched transcripts were higher for the 14FR vs 13FR (21 + 36), whereas there were zero enriched GO pathways. Could the authors comment on this? Were other pathways tested for?

In Fig 2C, the enriched pathways seem too general, and the authors do not follow up on any of them in more detail. For example, the data shows an enrichment for interferon beta response pathway. Can the authors show a longitudinal follow up of IFNb similarly to what is done on panel D of this figure. This would make the transition and connection to the longitudinal data in D more relevant and functional. As it is, the connection between transcriptomic analysis of whole colon tissue at 15 days of age and qPCR of "weaning reaction" cytokines when animals are on milk diet, transition to solid food and have started eating solid food is somewhat disconnected.

The data shown in Fig 2D decouples the effects dependent on the food vs. microbiota. However, the data could be presented in a clearer way to facilitate conclusions by the reader. Because GF mice do not experience the "weaning reaction" (increased Ifng, Tnfa), it is not clear how the comparison to GF mice at d15 post-birth adds to the story. Are the authors claiming that Ifng and Il17f are independent of the microbiota and dependent on fiber intake by the mothers? Can the data be displayed differently (perhaps using the same order as shown in Fig 3)?

In Fig 3, the comparison of the different immune cell subsets across different diets, in the presence and absence of microbiota, or specifically in the absence of AM, is a very interesting and critical approach. However, the data shown in this figure does not provide a clear dissection of the impact of diet versus microbiota on the immune parameters analyzed. For example, in panel A, ILC2 increases with fiber intake under 14SM condition, but decreases with fiber intake in GF condition, whereas no difference was observed in the absence of AM. What does that mean? Also, it looks like the data in panel C and D for groups 14SM FR and GF FR are the same shown in panels A and B which is not statistically sound (or recommended). How was the stat analysis performed? It also looks like the ANOVA tests were performed twice with the same data. The correct way is to add the 13SM FR data to the comparisons shown in A and B. If the authors wish to display the data in two separate graphs, they can indicate the values shown in A and B with dashed lines.

To better understand the implication of these immune cell alterations (and to narrow down which cell could potentially have functional outcomes), an immune cell characterization on adult mice (or even at d30 post-birth) that are fed either fiber free or fiber rich diets should be performed. Also, is the impact of fiber consumption on the immune system and microbiome composition permanent or transient? If mice are switched back to a fiber-rich diet after being born from dams fed a fiber-free diet, would they restore their immune and microbial compositions?

Referee #3 (Remarks for Author):

Grant et al. perform a gnotobiotic mouse experiment testing whether maternal diet or microbiome composition alter the colonic microbiome and physiology of pups during early life. They demonstrate that the maternal diet or the exclusion of a single species from a defined microbial community significantly changes the pups' colonic microbiome, transcriptome, and immune cell populations during the weaning period. Previous work examining the impact of maternal diet and microbiome on early life immune development largely focused on maternal high-fat diets and comparing germ-free to conventional mice. Thus, the experiment performed by Grant et al. provides additional support for the ability of maternal dietary fiber intake and a single gut commensal (A. muciniphila) in a defined community to alter offspring microbiome and intestinal immunity. This study appropriately describes the authors' findings, setting the stage for future work testing how the observed changes in the colon impact adult disease development and the mechanisms controlling infant microbiome composition before consumption of solid food. Given that maternal diet is typically modifiable and the microbiome can also be targeted, understanding how these two factors affect infant health is critical to developing optimal nutritional guidelines during pregnancy.

Major criticisms:
1. The most significant criticism is that the pups eat the same diet as the dams, thus making it difficult to distinguish (in particular during the later time points) whether the observed differences are driven by maternal diet, infant diet or copraphagy. The authors touch on this point in the conclusion in lines 186-189. However, because this is a key caveat, I think the authors should address this point much earlier in the manuscript and better describe their methods (when/are pups removed from the cages?)
2. The manuscript describes analysis of a large mouse experiment. Additional mouse experiments are not required. However, answers to the following questions would further strengthen the paper:
a. Were the litter sizes significantly different between breeders with different diets/microbiomes?
b. Were there any gross physiological differences between pups of the different breeders (e.g. differences in body weight, grooming, etc.)?
c. What is the sex of pups were used for the experiments and were there any differences in the data when segregating by mouse sex?
d. Can some of the top colonic enriched transcripts identified by RNA-Seq be validated by longitudinally (qPCR)?
e. Clarify if the selected cytokines differentially expressed at day 15 as measured by qPCR (Fig. 2D) were identified in the RNA-Seq dataset?
Minor criticisms:
1. Relative abundance (Figure 1 and Table S2-4):
a. How do the relative abundances by 16S rRNA sequencing (Table S4) compare to the relative abundances as determined by qPCR (Fig. 1, Tables S2-3)?
b. Please clarify in Fig 1B legend why some microbe names are bolded and some have asterisks.
c. The authors observed some differences in microbiome composition between 14SM FR and 13SM FR groups (e.g. M. formatexigens). Perhaps the authors should also describe these findings in their results?

2. Absolute abundance (Tables S2 and S3):
a. Please clarify what is meant by "Count."
b. What are the units for absolute abundances?
c. How were absolute abundances calculated?
d. Would it be possible to graph absolute abundances as in Figure 1C?
e. There is a wide range of total counts for absolute abundances (ranging from 55 to 34552 counts). How reliable are the relative

abundances determined from samples with very low total counts?

3. RNA-Sequencing (Fig. 2 and Fig. S1):
a. Fig. 2A: What does it mean for there to be no enriched GO pathways comparing the 14FR and 13FR transcripts? Does this mean there are no clusters of differentially expressed genes or that the functions of the differentially expressed genes are unknown?
b. Fig. 2C: The GO analyses for transcripts enriched in the 14FR group were not discussed in the manuscript. Also, how meaningful is to show these pathways considering they are based on only 2 differentially expressed genes?
c. Fig. 2D: Please provide the data (in the supplement) that Hprt was the most stable of the three housekeeping genes tested.

4. Model (Fig. 4):
a. The fiber-rich maternal diet model suggests that Akkermansia relative abundance is low at day 10, increasing to a peak at days 15-20, before decreasing to a stable abundance day 20+. However, the data the authors presented indicates that Akkermansia relative abundance is higher at day 10, then dips at days 15-20, before increasing to stable abundance at day 25 (Fig 1). Could the authors please clarify?
b. For the fiber-free maternal diet model, the authors not only saw an expansion of AM and BC, but also of DP and CS. Perhaps it is also important to include these organisms in the model?

5. Methods:
a. How long were the parental mice maintained on a standard rodent chow once they were colonized with bacteria and before being switched to their respective diets?
b. Were the male breeders maintained on the experimental diets or the standard diet?
c. When were the samples for parental microbiome composition analysis obtained and how were they collected?
d. How old were the pups when they were weaned?
e. How was the stream graph generated?

6. Text clarifications:
a. Title: The manuscript does not test how immune system responds in the pups. Perhaps a more appropriate title would be to change "immune responses" to "immune cell populations," "mucosal immunity," or "colonic immunity."
b. Line 27: Mice were fed fiber-free or fiber-rich diets.
c. Lines 37, 191, 626: I do not think the data can distinguish whether there are differences in immune priming or in recruitment of immune cell subsets. Perhaps the phrasing "early colonic immune cell populations" would be more appropriate?
d. Lines 71-73: The current phrasing implies that maternal diet primarily impacts the host epithelial transcriptome and that the presence or absence of A. muciniphila primarily impacts immune cell populations. However, the authors present data showing that both diet and A. muciniphila impact both host epithelial transcriptome and immune cells.
e. Line 86: Based on Tables S2 and S3, R. intestinalis and E. rectale are present in adult mice, but absence from pups.
f. Line 88: Did these bacterial populations significantly expand due to increases in their absolute abundance or decreases in the absolute abundance of other members of the microbiome?
g. Line 96: Please clarify if total RNA sequencing was performed on the total, proximal, or distal colon.
h. Line 128: The subtitle of this section is awkward.
i. Lines 142-155: This paragraph is not very clear. If the manuscript needs to be shortened, this paragraph could be cut. If the authors would like to keep this paragraph, please clarify to which metabolites the authors are referring (line 145), reference figures 2D and 3A at line 149, and rework the sentence in lines 150-153.
j. Lines 160-162: The "lower induction" and "lower repression" is a little confusing on a quick first pass. If the authors are able to rephrase the comparison to make it a little clearer it may be helpful.
k. Line 173: Please include "colonic" when describing the transcriptomic profile.
l. Line 195: Please add a "to" between "components" and "improve".
m. Line 577: Please add "targets" before "present once per genome."

7. Refences to include:
a. Line 64: The claim that A. muciniphila is capable of degrading milk oligosaccharides should also reference Luna et al (2022) (doi: 10.1128/AEM.01487-21).
b. Line 64: The claim that A. muciniphila is an early colonizer of the gut is supported by Collado et al (2007) (DOI: 10.1128/AEM.01477-07).
c. Reference list: Some of the citations in the reference list do not show the full list of authors.
d. The following articles may be important to mention when describing our current knowledge of whether maternal diet influences offspring immunity: Babu et al, (2018) (DOI: 10.1172/jci.insight.99223) and Thorburn et al, (2015) (doi: 10.1038/ncomms8320).

8. Miscellaneous:
The authors' suggestion that differences in IgG in breast milk may explain the differences in A. muciniphila abundance in pups (Line 198) is an intriguing idea, especially since mice can generate anti-Akkermansia IgG1 antibodies (doi: 10.1126/science.aaw7479).

***** Reviewer's comments *****
Referee #1 (Remarks for Author):

In this report, Grant and colleagues describe the evolution, in the intestine, of a consortium of 13 (no *Akkermansia muciniphila*, Am) or 14 bacteria (with Am) from age 10 days to age 30 days after birth. These mice are also given fiber-rich or fiber-free diets as an additional variable. RNA sequencing was performed on the colon of 15 days old mice, cytokines measured by qRT-PCR and different subsets of immune cells measured by flow cytometry.

This is an interesting study that yields many useful data. In particular, it analyzes the impact of Am not in isolation in monocolonized mice, but rather in the context of more complex microbiotas. But then, the data presented is complex, and it is difficult for the reader to connect all the dots. This is OK for cytokine measurements and cytometry of immune cells, but not OK for RNA sequencing data.
We thank the reviewer for highlighting the strengths of our study, particularly regarding studying the impact of *Akkermansia muciniphila* in the context of more complex microbiotas. We agree that the RNA-Seq data is complex and warrants further discussion. In our efforts to address this and similar comments made by the other reviewers, we have added a several points elaborating on the RNA-Seq data (lines 122-136, 144-150). We hope that the additional information facilitates readers' understanding of the data and its interpretation, but we welcome specific feedback on this point, in case questions remain.

Furthermore, I would suggest that missing is a metabolomic analysis of the output of the different microbiotas in the context of different diets. This may allow identifying compounds that act on immune cells and thus, for the authors to generate a more mechanistic set of data to link diet, microbiota and immunity.
While we agree that performing metabolomic analysis in young pups is of interest in order to identify potential microbial factors shaping differentiation of immune cells, it is technically challenging to obtain enough luminal contents from these animals (particularly pre-weaning) to be able to reliably perform metabolomic analysis (for just SCFA analyses, we would need at least 30mg of material). We attempted to enumerate differences in SCFA concentrations at 15 days of age, as this might help us to understand differences in Tregs, which were significantly higher in 13SM FR mice at 15 days of age, compared to the other colonized groups. However, as evidenced in the graph to the right, only 2 of the 20 samples reserved for this purpose met the minimum weight requirement for analysis by GC/MS, therefore we were forced to forego this analysis. We gratefully acknowledge the reviewer’s suggestion and would be open to troubleshooting with smaller weights in collaboration with the metabolomics platform.

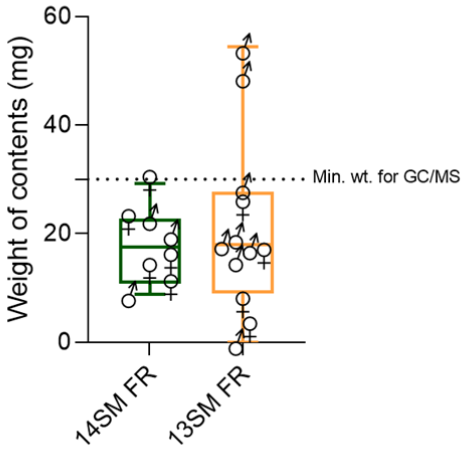

However, the underlying methodological optimizations would demand additional time investment beyond that required for animal ethical approvals (note that the ethical approval requires about 8 months in Luxembourg). Thus, given that the metabolic analyses are not essential to support the main conclusions of the current study, we do not find the repetition of the study and additional use of animals justified in this case. Futhermore, we have discussed this point with the editor, who noted “We also understand the challenges regarding the metabolomic experiment, but would like to see discussion regarding that point in the manuscript.” We agree with the reviewer and editor that this detail surrounding the metabolomic analyses is important, therefore we have added a couple of sentences to elaborate on the predicted metabolic changes based on the observed immune profiles.

“*Bacteria-derived metabolites represent a mechanistic link between changes in the microbiome and host immunity. While it would be highly interesting to perform metabolomics analysis during this early life period, we note that the overall volume of colonic contents available is a major limitation, especially at 10-15 days of age. In the absence of such data, we would speculate that microbial metabolism of tryptophan, which is abundant in milk, may drive many of the observed microbiota-mediated immune changes*.” (Lines 187-192).

A few comments:

1. Figure 2D: values for Tnfa, Il17f and Il22 are rather low and highly variable. Are different experiments, cages of sexes pooled? If yes, this should be indicated on the plots (using different symbols for example) in order to appreciate the source of variability. The statistical method should also be adapted to account for potentially linked datasets.
2. Figure 3A: similar comment. In addition, several data group are bimodal in distribution (ILC3 in particular). What is the source of bimodal distribution here, as it might explain apparent differences between groups?
Indeed, the data for *Tnfa*, *Il17f* and *Il22*, etc represent values for mice of each group from different experiments, cages, and sexes. Although it would be ideal to convey this metadata graphically, we find that doing so makes an already complicated figure more difficult to interpret, without contributing to or changing the interpretation. Therefore, we have elected to include the descriptive metadata on the cage, litter, and sex of mice in the accompanying source data spreadsheets (e.g. Source Data for **Fig 2D** and **Fig 3A**). There is no clear source for the observed variation; however, we note that these measures are taken during a very dynamic period, so a high degree of variation is not wholly unreasonable.

3. The data reported in Figure 2C is potentially interesting, but is left "hanging" in the study with no clear connections with the rest of the study (so not exploited). Some effort should be done to improve this issue.
Please note that we previously referenced **Fig 2C** in the text as **Fig 2B** (lines 121), which may have contributed to apparent lack of connection to the rest of the text. This typo has been corrected and we further expanded on the data to improve its integration with the rest of the study (lines 122-136).

Referee #2 (Remarks for Author):

Grant et al sought to study how fiber intake by the mother can imprint the immune system of the offspring, and how changes in the microbiota composition induced by the absence of fiber are involved in this process. They colonized mothers with a defined consortium of bacterial strains isolated from human feces and analyzed the impact of dietary fiber intake by mothers (and their pups) on the microbiota composition and on the development of the intestinal immune system at early life of the offspring. The authors propose that pups of fiber deprived dams display a delayed colonization of *Akkermansia muciniphila* (AM), and that removal of the same species under fiber sufficient state results in a decreased RORgt-positive innate and adaptive immune cells. They concluded that *A. muciniphila* contributed to an early-life immune imprinting. Although the authors are addressing an important biological question - related to the interplay between diet, microbiota and intestinal immune responses - with potentially interesting implications, the data lacked coherence. Whereas the read-outs for microbiota composition are clear and conclusive, the read-outs utilized to characterize the immune responses, and whether (and how) *A. muciniphila* impact the "weaning reaction" are less clear, and its implications less conclusive. Therefore, I recommend revision and addition of new experiments to support the author's conclusions. See comments below.

General comments:
The authors chose a human consortium based on their previous paper DOI:https://doi.org/10.1016/j.cell.2016.10.043. However, it is not clear whether this consortium recapitulates the broad spectrum of immune responses observed in SPF mice. Are these bacterium species normally found in the microbiota of SPF mice? This is important considering the alteration of immune pathways related to microbiota and fiber intake the authors propose in their study. How do the immune parameters evaluated in this study compare to WT SPF mice? Can the authors clarify and discuss these points?
We thank the reviewer for emphasizing this important point. Although the particular strains used in the 14SM model are not derived from mice originally, mice naturally harbor closely related strains and can therefore support colonisation with the 14SM community. We do not have reason to suspect that the fact that these isolates are human-derived would have a differential impact on the mouse immunity relative to mouse-derived isolates. Indeed, human microbiota-associated mice are frequently used to explore causal relationships on immune-associated readouts (DOI: 10.1126/scitranslmed.aab2271, 10.1126/scitranslmed.aaf6397, 10.1016/j.cell.2014.08.006, 10.1073/pnas.1711233114). Nonetheless, we acknowledge that such models also come with limitations. Regarding the point on the recapitulation of immune responses, we have indeed observed that SM-colonized adult mice do show some differences in proportions of certain immune cell populations compared to adult SPF mice (DOI: 10.21203/rs.3.rs-1563674/v2), as also observed in mouse-derived synthetic microbiota communities (DOI: 10.1016/j.chom.2022.09.011). Nonetheless, we note that 14SM mice represent an intermediate profile between SPF and germ-free conditions and, considering the importance of model tractability in exploring our research question, we find this to be a necessary and acceptable tradeoff. At the same time, we acknowledge that the reviewer raises some very important points that should be discussed further in the manuscript, therefore we have accordingly added a paragraph in Conclusion section to make the reader aware of this tradeoff:

"*While the 14SM model enables controlled investigation of the effects the microbiome on host immune development, it is important to note that the 14SM consortium is based on an adult human colonic microbiome. Although the species in this community are closely related to strains that are normally found in the mouse gut and thus readily colonise upon oral gavage in germ-free mice (Desai et al., 2016), the 14SM lacks certain members, such as bifidobacteria and lactobacilli, which are abundant in early life and mostly colonize the small intestine. These members could be added to the community in future studies, however we would also highlight that a community strictly designed to model the pre-weaning mouse gut*

would present its own set of limitations. To this point, Lubin et al. demonstrated that restricting the microbiome to a pre-weaning community halts immune development and increases susceptibility to Salmonella infection (Lubin et al., 2023), reflecting the need for a functionally-balanced synthetic community to elicit normal immune development. As with other gnotobiotic models, the 14SM immune profiles typically represent an intermediate between germ-free and SPF colonization states in adulthood, potentially due to low diversity, low bacterial density and/or absence of specific immunogenic bacteria such as segmented fillamentous bacteria (Parrish, Grant, et al., 2022). Addition of new members may be a promising approach to refine these models, although it remains difficult to completely recapitulate SPF immune profiles (Afrizal et al., 2022). Despite these limitations, synthetic microbiota models remain critical tools to dissect specific interactions between the microbiome and diet in early life" (Lines 284-301).

In the experimental setups used throughout the study, dams and pups are kept on a fiber-free or rich diet for the duration of the experiment. Even though the immune analyses are performed at early time points (presumably before the animals have started eating solid food), this could have a direct long-term impact on microbiota composition and intestinal immune system. To definitively conclude that the fiber intake by the mothers affect the immune system of the offspring, the pups born from mothers fed a fiber-free diet should also be given a fiber-rich diet at weaning age (d17-21 post-birth), and analyzed at d30 post-birth (same time point as the microbiota composition analysis). In other words, are the immune effects related to fiber intake by the mothers transient or long-term (see also specific comments for figure 3)?
While we agree that such a crossover set up would be interesting in providing a better understanding of the timing, we are skeptical that the results of such an experiment would be definitive towards answering the points raised by the reviewer. As the reviewer mentions, the key time point of interest occurs at 15 days of age, before the pups have transitioned to solid food. This timing strongly suggests that the maternal fiber intake drives the results observed in the present study. Further, we would argue that the ideal controls to understand the effects of diet versus microbiota are germ-free animals fed both diets, which we have carefully analysed in our study.

We would like to point out that we would require additional ethical approval for such an experiment, which would take about 8 months in Luxembourg, including approval from the Ministry of Health. It would additionally take another 6 months or so to perform the experiment and analyse the data. Given such practical limitations, timeliness of our study and the fact that this particular experiment would likely yield incremental results in the context of the current study, we think that the suggestions of R3 (i.e. to more critically discuss our results and specific limitations of the model and experimental setup) are more practical in order to re-submit the manuscript within the stipulated time frame. We have therefore openly discussed these options with the editor, who agreed that additional animal work is not needed, but that, in line with the suggestions of R3, we critically discuss our results to better address the questions raised by the reviewer.

The suggestion regarding long-term fiber intake versus transient fiber intake (switching FF dams to a FR diet) is an interesting approach, but which we feel is beyond the scope of this short report. Additionally, the immune analysis in 30-day-old animals nursed by FF dams and weaned onto the FR diet would not help to further dissect the effect of diet versus microbiota on the immune system in 15-day-old pups and subsequent susceptibility to disease in adulthood. Such a question would require implementation of a disease model and, although it is important to understand how the early life window shapes the adult immune system, such experiments are beyond the scope of the current study. Certainly, we would encourage and highlight the need to investigate such questions through subsequent experiments, therefore we have integrated this suggestion into our discussion:

"*In this study, we assessed the compounding effects of a fiber-free diet across generations with mice pups weaned onto the same diet as the dams. While the observed immune changes occur prior to weaning, modifying the diet at distinct developmental stages (in utero, pre-weaning, and post weaning) would provide insights into critical key drivers of early life immune development. Furthermore, it would be of interest to interrogate both the persistence of the observed differences in early life immunity in an adult mouse and the effects of this differential development in a relevant disease model (**Fig 4**). For example, Al Nababi et al. showed that the weaning reaction provided protection against oxazolone-induced colitis—a type-2 inflammation—and, considering the enrichment type 2 immune cell subsets in pups of 13SM FR-fed mice at 15 days of age, this could be an interesting model to further investigate diet and microbiota effects on immunity at distinct developmental stages. Thorburn et al., for example, found that fiber supplementation during pregnancy alone was sufficient to prevent asthma development in offspring (Thorburn et al, 2015). The impact of the duration and timing of a fiber intervention in early life and the implementation of a disease model would both be clear points to investigate in subsequent research. Although the involvement of the microbiome was not explored by Thorburn et al., we previously documented the synergistic impact of diet and* A. muciniphila *in the context of food allergy (Parrish et al, 2022a). However, the study by Parrish et al. was performed in adult mice that were colonized by oral gavage, therefore the question remains as to how exposure to* A. muciniphila *in early life might play into the development and severity of allergic responses. Finally, mouse models of multiple sclerosis might also be of interest considering RORgt+ immune cell subsets were strongly affected by the maternal diet or microbiome changes in the present study and this same subset is also known to drive the demyelination that characterizes this disease.*" (Lines 244-263).

Specific comments:
In Fig 1A, there appears to have a typo in the experimental scheme. It should be 13SM fiber-rich instead of fiber-full.
Thank you for catching this error. We confirm that the 13SM mice were fed a fiber-rich diet, not a fiber-free diet and have corrected the figure key.

In Fig 1C, what are the implications for the changes in microbiota composition (besides AM) over time comparing fiber-free and fiber-rich diets? For example, what does the change in Dp or Bc mean?
We appreciate this comment and have added additional explanations regarding the role of these species in host health as well as the implications of their elevated abundance under fiber-deprived conditions.

"*As* B. caccae *can degrade complex polysaccharides and mucin, its elevated abundance on the fiber-free diet may reflect a functional shift towards increased mucin foraging, which can ultimately contribute to thinning of the mucosal barrier (Desai et al, 2016) and promote production of immunostimulatory cytokines TNF and IL-6 (Brown et al, 2021). Although the physiological consequences of an increase* C. symbiosum *are less clear from a functional standpoint, it is elevated in IBD and is an emerging biomarker for colorectal cancer (Xie et al, 2017). Similarly,* D. piger *may also contribute to development of local inflammation and colorectal cancer via production of hydrogen sulfide (Blachier et al, 2021), particularly when combined with increased liberation of sulfate moieties by mucin-foraging bacteria under fiber-free conditions.*" (Lines 88-97)

In Fig S1, is the variance on PC1 due to batch effect (14FR1 and 14FF4 compared to rest)? Also, this PCA plot shows that groups are comparable (no major differences between the groups), could the authors comment on this?
Although the PCA is based on the top 500 most variable genes, on a global level, it appears that the differences between the groups are limited to a small fraction on the transcriptome. Indeed this data is reflected in the relatively small number of DESeq2 results of transcripts

that are up/down in pairwise comparisons (for comparison, in an adult 14SM model with worm infection sequenced at the same time there were nearly 300 transcripts that were differentially expressed between FR and FF mice; DOI: 10.1101/2022.02.28.482289). Please also note that the inclusion of the outliers only slightly changes the number of transcripts that were significantly up or down in the 14SM FR group relative to 13SM or FF:

DESeq2 results		13FR vs 14FR	14FF vs 14FR
All samples	up in 14FR	18	31
	down in 14FR	21	7
Outliers 14FR2 and 14FF4 removed	up in 14FR	21	25
	down in 14FR	36	7

All samples were sequenced at the same time, however 14FF4 was from a different litter than the other 3 in that group and 14FR1 was from a different cage, therefore it is possible that these differences are reflected in a more distinct transcriptomic profile. We surmise that batch effects can be a plausible explanation for the difference in the 14FR1 and 14FF4 samples, therefore we elected to exclude them on the basis of this factor and the PCA.

The model in Fig 4 shows that under control condition (14SM, Fiber rich diet), AM expands and shrinks over the weaning period. However, the data in Figure 1B (middle panel) and 1C (AM panel) does not show clearly whether AM is significantly different across the different ages of the pups. It looks like D20 and D30 are very similar in fiber-free diet group whereas D15 and D30 are very similar in the fiber-rich diet. This is particularly important because at D20 the pups have started eating solid food. Can the authors explain this?
In our summary figure (**Fig 4**), we attempted to convey that the starting abundance of AM would have been zero, but we understand that this was poorly aligned with the age of the mice as we start the timeline at 10 days of age. In the updated **Fig 4**, we have adjusted this line to match more closely the observed abundances, beginning at 10 days of age. Regarding the point about comparing the change in abundances within a group, we agree that the reviewer makes a valid point to consider the impact of the sampling day. However, after correction for multiple comparisons, only the 14SM FF group had significant changes in AM abundance over time (day 10-15 versus day 20-30), in alignment with the pre- and post-weaning periods. In a 2-way ANOVA analysis, the group (in this case, the fiber-rich or fiber-free diet) and the interaction between group and day each contributed to over 1/3 of the observed variation, while the sampling day alone explained roughly 10%. So while the day is not at all unimportant, the influence of diet on this dynamic seems to be of greater relevance. Regarding the second point that "D20 and D30 are very similar in fiber-free diet group whereas D15 and D30 are very similar in the fiber-rich diet", there are two considerations to keep in mind. The first is that the compositional data is proportional and an expansion in another taxa at day 20 (potentially CA or BO in 14SM FR group), can result in an apparent decrease in AM. Whether AM is actually shrinking at that timepoint is less clear. Likely, as the reviewer points out, the timing of the weaning plays some role here and the shrinking proportion reflects an expansion of other fiber-degrading taxa as the pups begin to consume solid food.

In Fig 2, how do the authors reconcile the differences in mRNA expression of weaning reaction cytokines (Ifng and Tnfa) in the colon of mice described in this study compared to the study of Al Nabahani et al, 2019 in which the authors show increase mRNA expression of ifng and Tnfa in the ileum of SPF mice at D21 but not at D15 post-birth? Can the authors explain why D20 in the present study is similar to D10 post-birth but elevated at D15 post-birth?
The Swiss Webster mouse strain used in our study is slightly larger and matures faster than the C57BL/6 strain, which was used in the Al Nabhani paper. To illustrate this, Swiss can be separated from the mother at 3 weeks of age, whereas C57BL/6 generally are not separated until 4 weeks of age (also in Al Nabhini paper). The timing of the reaction seems to coincide with gradual transition to solid food and resultant expansion of diverse immunostimulatory members of the microbiome, therefore differences in the onset of this process would be

expected to reflect differences in the mRNA expression of Tnfa and Ifng. Indeed a 3 week-old Swiss Webster pup is approximately the same weight as a 4 week-old C57BL/6 pup, suggesting that the activity level and ability to reach and consume solid food may arise earlier in Swiss mice and therefore bring about an earlier "weaning reaction" than what was observed in C57BL/6 mice.

In addition, how the authors explain the role of diet on the cytokine mRNA expression in germ-free mice since they are not exposed to microbiota during the weaning period and therefore do not experience the so-called "weaning reaction"?
The question of the role of diet on the cytokine mRNA expression in germ-free mice is indeed a very interesting point that we agree should be elaborated on in the manuscript. We have added the lines 164-166 to the existing explanation in lines 159-163 accordingly:

"*By comparing transcript expression levels to germ-free (GF) mice at age 15 days (**Fig 2D**), we see that some of the observed pro-inflammatory shifts can be attributed to a combination of diet and microbiome composition. In particular, at age 15 days, the FF diet increased the transcript levels of* Ifng, Il17f, *and* Il23 *among germ-free pups of fiber-deprived dams, relative to germ-free, fiber-rich-fed offspring (**Fig 2D, Fig EV1C**). Although the expression of these cytokines is overall lower in germ-free conditions compared to 13SM or 14SM, differences in the maternal diet may affect the baseline expression of these cytokines in germ-free mice through a different maternal milk composition*".

Were the outliers removed for statistical analysis but included in the graph?
Plots were generated from "cleaned" datasets in GraphPad, that is, following the removal of outliers identified according to the ROUT method (Q=10%).

Since mRNA expression by qPCR does not follow a linear distribution, were the data log-transformed prior to stat analysis?
We have log-transformed values prior to statistical analysis and can confirm that the residuals follow a normal/Gaussian distribution according to the Kolmogorov-Smirnov test. This explication has been added to the "Statistical analysis" section under Materials and Methods.

In Fig 2A, it is surprising that the number of enriched transcripts were higher for the 14FR vs 13FR (21 + 36), whereas there were zero enriched GO pathways. Could the authors comment on this? Were other pathways tested for?
We agree with the observation that it is surprising that, despite a higher number of differentially expressed transcripts, there were no differences on a functional/gene set level. The methodology for testing was identical between the two comparisons. That is, we passed the data for the transcripts which were differentially enriched to the enrichGO() function, which evaluates whether the number of genes/transcripts belonging to a given GO class are over-represented. It is worth noting that this analysis is on a functional level, to characterize similar processes that are mutually affected, but not necessarily part of the same biological pathway. Although these terms are often used interchangeably in the literature, we have substituted "pathway" for "gene set", where appropriate to avoid confusion regarding the interpretation of this data. We also comment on this aspect at greater length in the revised manuscript:

"*Performing a gene set analysis based on Gene Ontology (GO) terms for biological processes, we found differential enrichment according to the diet (14SM FR vs 14SM FF), but not to the presence of* A. muciniphila *(14SM FR vs 13SM FR) (**Fig 2A bottom, Fig 2C**). This may reflect two limitations of gene set analyses: 1) transcripts not corresponding to a common pathway can still directly lead to changes in immune cell differentiation and 2) transcripts corresponding to pseudogenes or ncRNA may impact on host development through epigenetic mechanisms or other undescribed pathways (**Fig EV1B, Dataset EV6**).*

*Such cases would not be highlighted in gene set analyses, therefore, while gene set analyses can be quite powerful to highlight common functional changes, the absence of significant changes on the level of gene sets for the 14SM FR vs 13SM FR comparison does not necessarily mean that there are no relevant differences in the transcriptomes. For example, H2-K1 (a histo-compatibility gene involved in antigen presentation), Gapdh (involved in glycolysis), and Gal3st2b (involved in glycosylation) represent relevant differences between the 13SM and 14SM groups that can underlie the observed immunological differences, but which do not correspond to a shared functional pathway (**Dataset EV6**)*. (Lines 119-132).

In Fig 2C, the enriched pathways seem too general, and the authors do not follow up on any of them in more detail. For example, the data shows an enrichment for interferon beta response pathway. Can the authors show a longitudinal follow up of IFNb similarly to what is done on panel D of this figure. This would make the transition and connection to the longitudinal data in D more relevant and functional. As it is, the connection between transcriptomic analysis of whole colon tissue at 15 days of age and qPCR of "weaning reaction" cytokines when animals are on milk diet, transition to solid food and have started eating solid food is somewhat disconnected.
We thank the reviewer for this insightful comment. For the revised version of this manuscript, we have designed and tested primers targeting the differentially enriched transcripts belonging to the gene sets identified as up or downregulated. The longitudinal results align with the RNA-Seq data, although the groupwise comparisons were not significant for all transcripts. These data are presented in **Figure EV1**.

The data shown in Fig 2D decouples the effects dependent on the food vs. microbiota. However, the data could be presented in a clearer way to facilitate conclusions by the reader. Because GF mice do not experience the "weaning reaction" (increased Ifng, Tnfa), it is not clear how the comparison to GF mice at d15 post-birth adds to the story. Are the authors claiming that Ifng and Il17f are independent of the microbiota and dependent on fiber intake by the mothers? Can the data be displayed differently (perhaps using the same order as shown in Fig 3)?
We understand the reviewer's concern. However, we would like to point out that this request is in slight contradiction with the next request, where the reviewer critiques the splitting of data in **Fig 3**. After careful deliberation, we agree that it is important to present the groups in a consistent ordering and also to keep all groups together in order to not repeat datasets, as discussed below for the next point. Therefore, in order to address the points raised by the reviewer, we have rather aligned the data presented in **Fig 3** with that on **Fig 2**, for consistency and statistical soundness. Indeed, as the reviewer points out, germ-free mice do not undergo a "weaning reaction" (as defined by the induction of Ifng and Tnfa, according to Al Nabhani et al.). Nonetheless, the germfree pups born to dams fed a FR or FF diet represent an important control, as we would predict that the FF diet can elicit a distinct host response even in the absence of the microbiome. It is important therefore to be able to compare to such a non-colonized control group and identify transcriptional changes associated with the FF diet alone.

In Fig 3, the comparison of the different immune cell subsets across different diets, in the presence and absence of microbiota, or specifically in the absence of AM, is a very interesting and critical approach. However, the data shown in this figure does not provide a clear dissection of the impact of diet versus microbiota on the immune parameters analyzed. For example, in panel A, ILC2 increases with fiber intake under 14SM condition, but decreases with fiber intake in GF condition, whereas no difference was observed in the absence of AM. What does that mean? Also, it looks like the data in panel C and D for groups 14SM FR and GF FR are the same shown in panels A and B which is not statistically sound (or recommended). How was the stat analysis performed? It also looks like the ANOVA tests were performed twice with the same data. The correct way is to add the 13SM

FR data to the comparisons shown in A and B. If the authors wish to display the data in two separate graphs, they can indicate the values shown in A and B with dashed lines.
We appreciate the reviewer's suggestions to help us present the data in a more convincing way. We confirm that the data in panel C and D for groups 14SM FR and GF FR are the same shown in panels A and B. We originally chose to split the data to answer two distinct questions: 1) effects of diet and colonization in panels A/B and 2) the effects of *Akkermansia* and colonization in panels C/D. We felt that splitting the presentation in such a way would be beneficial to support the reader since there are a number of comparisons being made. However, in light of the suggestion above for **Fig 2** and the considerations for the statistical analyses, we revised the figure and opted to present the data for all groups in a single plot, in line with how we have presented the transcript data.

We have added a discussion of the point regarding raised for ILC2s, which we attribute to a combination of 3 factors: the diet, colonization, and proportional nature of the immune datasets. "*Under FR conditions, colonization differentially affected the proportions of innate cell subsets, with ILC3s favored under 14SM conditions (p=0.0023) versus ILC2 cells under 13SM conditions (p=0.0400) (**Fig 3A**). Although the FF diet skewed toward ILC2 in the absence of a microbiota, the strong enrichment of ILC3s in 14SM colonized mice (p=0.004) led to a null effect when comparing ILC2 proportions in pups of 14SM and GF FF-fed dams directly. These results highlight the complex interactions between diet and microbiota on immune development in early life.*" (Line 206-212).

Regarding the statistical analyses, we value the critical input by the reviewer regarding the best practices. We have revisited the analysis of our data and systematically introduced outlier detection by ROUT method (Q=10%), followed by a test for normality on the cleaned data, and finally a one-way ANOVA with correction for all possible groupwise comparisons using the BH method. The statistics shown are adjusted for all group comparisons, with adjusted p-values displayed only for the biologically relevant group comparisons, as explained in the "Statistical analysis" section. Please note that this resulted in some changes to the adjusted p values.

To better understand the implication of these immune cell alterations (and to narrow down which cell could potentially have functional outcomes), an immune cell characterization on adult mice (or even at d30 post-birth) that are fed either fiber free or fiber rich diets should be performed. Also, is the impact of fiber consumption on the immune system and microbiome composition permanent or transient? If mice are switched back to a fiber-rich diet after being born from dams fed a fiber-free diet, would they restore their immune and microbial compositions?
We agree with the reviewer that immune characterization in the adult mouse would be a logical next step in this work. We hope to integrate this analysis into a larger project also including a disease model, however we felt it was prudent and timely to already share the data we have generated in this project in order to highlight compelling avenues of research for others in the field as well. As explained above in detail, given the short report format, we find additional animal experiments would substantially delay the dissemination of these early results. We have incorporated the reviewer's recommendation into our conclusion section in order to explicitly define the areas where follow-up studies are needed: "*Furthermore, it would be of interest to interrogate both the persistence of the observed differences in early life immunity in an adult mouse and the effects of this differential development in a relevant disease model ...* " (Lines 248-263). One important point here is also that it would likely not be sufficient to assess the immune profiles in adult models. An additional trigger, through induction of disease, for example, would be critical in order to properly flesh out the consequences of these early life differences in immune priming.

Referee #3 (Remarks for Author):

Grant et al. perform a gnotobiotic mouse experiment testing whether maternal diet or microbiome composition alter the colonic microbiome and physiology of pups during early life. They demonstrate that the maternal diet or the exclusion of a single species from a defined microbial community significantly changes the pups' colonic microbiome, transcriptome, and immune cell populations during the weaning period. Previous work examining the impact of maternal diet and microbiome on early life immune development largely focused on maternal high-fat diets and comparing germ-free to conventional mice. Thus, the experiment performed by Grant et al. provides additional support for the ability of maternal dietary fiber intake and a single gut commensal (*A. muciniphila*) in a defined community to alter offspring microbiome and intestinal immunity. This study appropriately describes the authors' findings, setting the stage for future work testing how the observed changes in the colon impact adult disease development and the mechanisms controlling infant microbiome composition before consumption of solid food. Given that maternal diet is typically modifiable and the microbiome can also be targeted, understanding how these two factors affect infant health is critical to developing optimal nutritional guidelines during pregnancy.

Major criticisms:

1. The most significant criticism is that the pups eat the same diet as the dams, thus making it difficult to distinguish (in particular during the later time points) whether the observed differences are driven by maternal diet, infant diet or copraphagy. The authors touch on this point in the conclusion in lines 186-189. However, because this is a key caveat, I think the authors should address this point much earlier in the manuscript and better describe their methods (when/are pups removed from the cages?)

While our readouts cover the span of the weaning period, we largely focus on 15 days of age, just before the pups begin to consume a solid food diet. Between 15-21 days of age (pups weaned at 21 days, as now indicated in Methods and **Fig 1A**), pups may start to nibble on the solid food, but at 15 days of age, the pups are almost exclusively suckling, therefore the direct effect of the solid diet on the pups is negligible to the findings of this study. We note that the stools of pups are also not well-formed at 15 days of age, indicating that they are still suckling. Although studies on the onset of coprophagic behavior of pups are limited, one review reported this behavior is typically observed at 17-18 days of age among ICR mice (an outbred Swiss strain with a similar growth pattern to the Swiss Webster strain used in this study), after they begin excreting feces (Ebino, 1993; https://www.jstage.jst.go.jp/article/expanim1978/42/1/42_1_1/_pdf). We agree with the reviewer that future studies should investigate variations of the protocol employed here, as cross-fostering pups born to FR-fed dams with FF-fed dams (and vice versa) would help isolate the effects of the milk versus the in utero period, however this work falls beyond the scope of this brief report. In the absence of performing this additional experimental work, we agree completely that the study design and its limitations should be conveyed clearly in order to appease readers expecting a cross-fostering configuration or similar. Therefore, we have taken measures to elaborate on these points in the Materials and Methods sections “Mice” and “Diet formulation and administration” (lines 332-343).

2. The manuscript describes analysis of a large mouse experiment. Additional mouse experiments are not required. However, answers to the following questions would further strengthen the paper:

a. Were the litter sizes significantly different between breeders with different diets/microbiomes?

We found no significant difference between the 13SM FR, 14SM FR, 14SM FF, GF FR, or GF FF groups according to litter size. We have added a brief note in the manuscript accordingly under the "Mice" section of Materials and Methods (lines 330-332).

b. Were there any gross physiological differences between pups of the different breeders (e.g. differences in body weight, grooming, etc.)?
Although the body weights did not significantly differ between groups at the first and last time points, we do note some variations in pup weight by diet or microbiota during the weaning period. We have added data on body weight of each mouse to the EV2-3 Datasets and also included the comparisons as a new Extended View figure (**Fig EV3**), also below. We unfortunately do not have any data on grooming behavior, but will certainly keep this suggestion in mind for future studies in the early life model.

c. What is the sex of pups were used for the experiments and were there any differences in the data when segregating by mouse sex?
As with the previous suggestion (2b), we have included data on the sex of the pups in the EV2-3 Datasets.

d. Can some of the top colonic enriched transcripts identified by RNA-Seq be validated by longitudinally (qPCR)?
We have performed RT-qPCR on top differentially enriched transcripts from 13SM FR, 14SM FR, and 14SM FF groups at 10, 15, and 20 days of age as well as 15 days of age for GF FR and GF FF mice. We focused our efforts on transcripts belonging to gene sets that were altered, including *Ifnb1*, *Fos*, *Tgtp2*, *Igtp*, *Gbp2*, *Itga5*, *Clca3a2*, *F13a1*, *Nox1*, *Hspb2*, *Ppp3r2*, *Nlrp1b*, and *Ifitbl1*. The new datasets can be found in **Fig EV1**. In general, we found the trends to be consistent with expectations from the RNA sequencing, however the observed differences by RT-qPCR were not always statistically significant as the results by RT-qPCR showed more within-group variation than by RNASeq.

e. Clarify if the selected cytokines differentially expressed at day 15 as measured by qPCR (Fig. 2D) were identified in the RNA-Seq dataset?
For Tnfa, transcript levels in the RNA sequencing data were higher for 14SM FF on average, however this difference was not statistically significant, owing in part to both the low power (just 3-4 mice in RNA-Seq versus >10 for RT-qPCR) and high variability for this transcript. The Il22 transcript was not identified in RNA sequencing mapping, possibly indicating insufficient sequencing depth for this transcript. Although the RNA sequencing and RT-qPCR approaches should be concordant (and indeed we see similar trends in our added validation assays; **Fig EV1**), the exceptions for these transcripts reflect limitations or caveats for each approach.

Minor criticisms:
1. Relative abundance (Figure 1 and Table S2-4):
a. How do the relative abundances by 16S rRNA sequencing (Table S4) compare to the relative abundances as determined by qPCR (Fig. 1, Tables S2-3)?
We have made some sanity checks between the two datasets, which we have pasted below. Except for *Clostridium symbiosum*, *Collinsella aerofaciens* and *Desulfovibrio piger*, the correlation coefficient between qPCR (y-axis) and 16S rRNA gene sequencing (x-axis) relative abundances was strong ($R^2 > 0.7$). We would suspect that the discrepancy in results for the three bacteria with weak correlations may be due to the low abundance of these bacteria in each sample, PCR biases, and/or 16S rRNA gene copy number biases. The 16S rRNA gene sequencing approach is not ideally suited to quantify abundances for the 14SM community because it is difficult to differentiate the three *Bacteroides* species. Additionally, there is the aforementioned issue of variations in 16S copy number, which also affects our SM community (16S rRNA gene copies ranging from 1 to 9). It is for this reason that we prefer to rely on the qPCR quantification, which targets genes that are unique to each strain and also present once per genome. The 16S data is largely to rule out contamination with non-14SM species, as described. These data have been added to the manuscript as Extended Data (**Fig EV4**). In our previous study (DOI:

https://doi.org/10.1016/j.cell.2016.10.043), we also evaluated the relative abundances with both 16S rRNA gene sequencing and phylotype-specific qPCR primers in adult mice and found similar results.

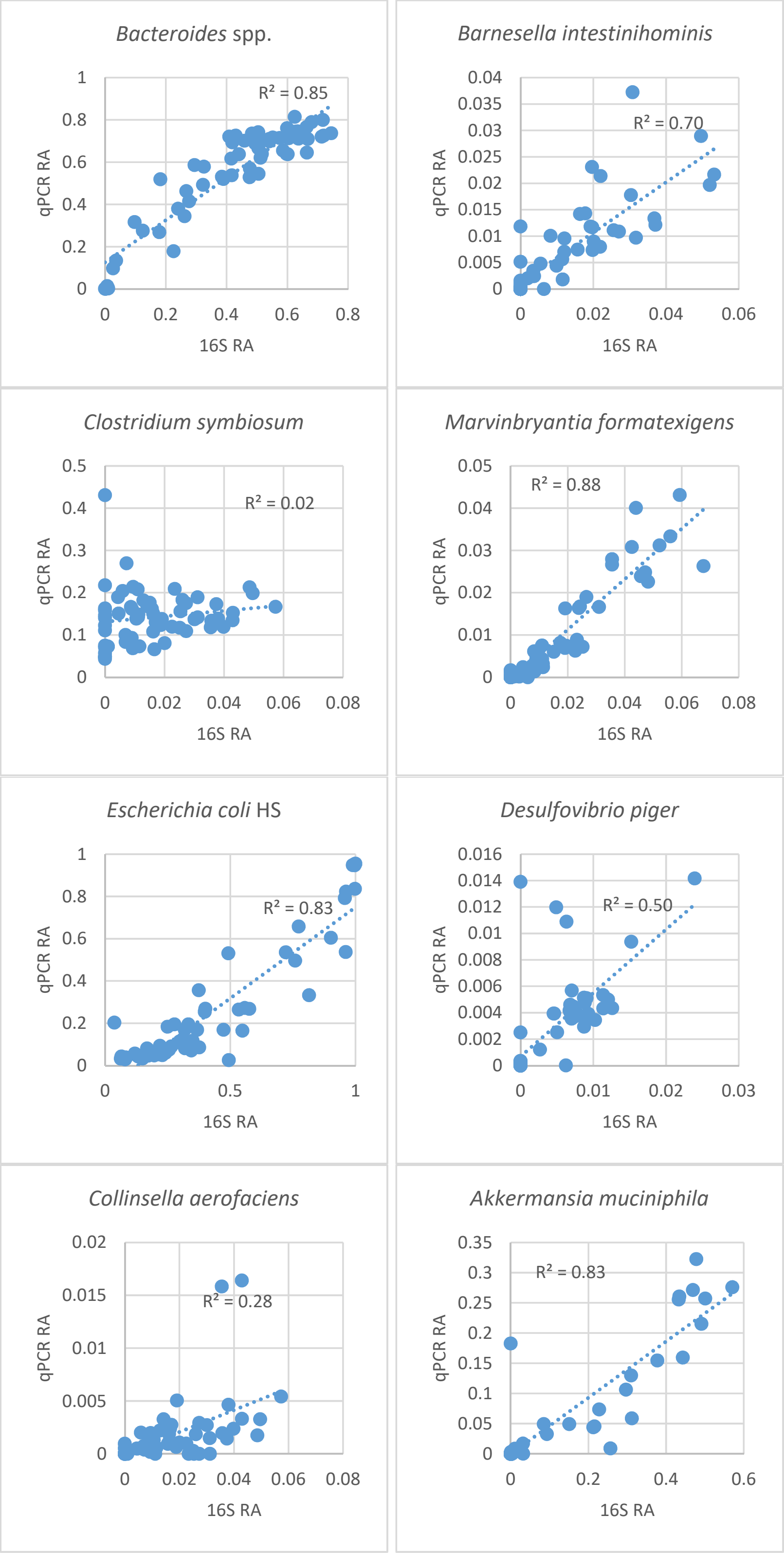

b. Please clarify in Fig 1B legend why some microbe names are bolded and some have asterisks.

This detail has been clarified in the legend. The names that were bolded corresponded to mucin-degrading taxa, which we highlighted due to the expansion of AM and BC in pups of FF-fed dams following weaning. However, we felt that the bolding was not necessary since this is discussed in the text, so we elected to remove this feature. The taxa with asterisks were present at <0.1% on average and we suspect were not well transferred from the parental mice. We note that in a similar experimental set-up (DOI: 10.1101/2022.04.03.486886; Figure 6J in this preprint) we likewise could not detect certain taxa pre-weaning, but these bacteria later expand in adulthood, suggesting that their abundance is too low to be detected by qPCR, but not necessarily that they are absent entirely. Nonetheless, this is why these taxa were also omitted from the longitudinal depiction in **Fig 1C**.

c. The authors observed some differences in microbiome composition between 14SM FR and 13SM FR groups (e.g. M. formatexigens). Perhaps the authors should also describe these findings in their results?

We appreciate this comment and have added additional explanations regarding the role of M. formatexigens and other species that were differentially abundant in the 13SM condition. "*Differences in the microbial assemblage between pups of 14SM and 13SM dams were less pronounced than between pups from FR and FF-fed dams. However, in pups of 13SM dams, we note a transient increase in the relative abundance of* Barnesiella intestinihominis*, a mucin-specialist, at day 25;* Collinsella aerofacians *at day 30, a bacterium linked to increased gut permeability; and stalled growth in* Bacteroides ovatus *at day 20, a bacterium linked to production of short-chain fatty acids and neuroactive compounds (Horvath et al, 2022). After weaning, there was also a marked increase in* Marvinbryantia formatexigens*, an oligo-saccharide degrading bacterium (Rey et al, 2010) that can stimulate host IgA production via acetate (Takeuchi et al, 2021). Although preliminary, these data indicate that the absence of* A. muciniphila *may have a distinct impact the community structure and host immunity through diet-independent mechanisms.*" (Lines 99-108)

2. Absolute abundance (Tables S2 and S3):

a. Please clarify what is meant by "Count."

A count is a typical generic term referring to one genome equivalent. Comparable terms are CFU (in culturing) or event (in flow/mass cytometry). The count refers to the calculated starting quantity (from qPCR), scaled by the total amount of gDNA extracted from the contents (i.e. per organ). Unfortunately, it was not possible to normalize these counts by the weight of the contents, as would typically be done, since the quantity was extremely low at days 10–15 and the collection protocol was adapted to maximize content recovery by washing the tissue in PBS.

b. What are the units for absolute abundances?

Kindly refer to the response above. In case this explanation is not clear, we are open to alternative suggestions.

c. How were absolute abundances calculated?

In adult mice, we could consider counts normalized to fecal weight, for example, however in this experimental set up, we washed the tissues in 1X PBS in order to maximize recovery of the contents for mice with minimal material in their colons. We have attempted to perform an assessment of absolute abundance by normalizing to the amount of DNA extracted, however this is also highly variable across samples and depends on the mass of the contents, therefore we elected to perform our statistical calculations using relative abundance measures.

d. Would it be possible to graph absolute abundances as in Figure 1C?
For the reasons discussed in the next point, we prefer not to graph the absolute abundances in the main text, however they are already plotted within the Dataset EV2.

e. There is a wide range of total counts for absolute abundances (ranging from 55 to 34552 counts). How reliable are the relative abundances determined from samples with very low total counts?
Indeed, the enumeration of absolute bacterial counts in early life is a major challenge, as discussed above, in response to point 2a. While we agree there is a large variation in the interpolated absolute abundances, the culprit for these difference lies in two key factors: the total amount of contents from which DNA was extracted and the age of the mouse (which should relate to the degree of exposure to microbes and ability of the diet to support specific taxa). The combination of these two factors, e.g. a young mouse with a minute quantity of available contents, can yield a low total count as observed. We have added this clarifying detail to the Materials and Methods: "*As tissues were washed in 1X PBS to collect the contents, this estimation is based on the total contents, not normalized by the content weight, and can therefore vary according to factors such as mouse age, diet, and recent defecation*." (lines 401-404). While we report the absolute abundances according to total contents, we expect that the relative abundances more reliably reflect the status of the colonization as long as we bear in mind that the total bacterial load is also increasing over time.

3. RNA-Sequencing (Fig. 2 and Fig. S1):
a. Fig. 2A: What does it mean for there to be no enriched GO pathways comparing the 14FR and 13FR transcripts? Does this mean there are no clusters of differentially expressed genes or that the functions of the differentially expressed genes are unknown?
Thank you for bringing up this very valid point, which was also raised by reviewer #2. As indicated in the volcano plot in **Fig EV1B** and **Dataset EV6**, there were numerous transcripts that differed between the two colonization states, however, many of these transcripts correspond to pseudogenes or ncRNA (beginning with "Gm" or ending with "Rik"). Such transcripts may have relevance on epigenetic level, but would not be expected to contribute meaningfully in a gene set level analysis due to poor annotation. We have added a note on this caveat to the manuscript: "*This may reflect … shared functional pathway (**Dataset EV6**)*." (Lines 122-132).

b. Fig. 2C: The GO analyses for transcripts enriched in the 14FR group were not discussed in the manuscript. Also, how meaningful is to show these pathways considering they are based on only 2 differentially expressed genes?
We agree that the changes in the 14SM FR group are less impactful than those for the FF group as the findings are based on just two genes. Nonetheless, the data should be given fair discussion alongside those gene sets that were elevated in the 14SM FF group. We have added additional discussion accordingly (line 134-136).

c. Fig. 2D: Please provide the data (in the supplement) that Hprt was the most stable of the three housekeeping genes tested.
We have added the RefFinder results under Dataset EV8.

4. Model (Fig. 4):
a. The fiber-rich maternal diet model suggests that *Akkermansia* relative abundance is low at day 10, increasing to a peak at days 15-20, before decreasing to a stable abundance day 20+. However, the data the authors presented indicates that *Akkermansia* relative abundance is higher at day 10, then dips at days 15-20, before increasing to stable abundance at day 25 (Fig 1). Could the authors please clarify?
We apologize for any confusion from this graphical illustration of our findings. Indeed reviewer 2 made a similar observation. We attempted to convey in both conditions a starting

abundance of 0%, considering the bacterium is not present upon birth, however we realize that the timeline at the bottom is incongruous with this aim. We have adapted the **Fig 4** so that the illustration more closely reflects the actual data as presented in **Fig 1B**.

b. For the fiber-free maternal diet model, the authors not only saw an expansion of AM and BC, but also of DP and CS. Perhaps it is also important to include these organisms in the model?
We have added additional text discussing the potential implications of changes in the abundances of these bacteria and also added these taxa to the graphical representation (**Fig 4**) as suggested.

5. Methods:
a. How long were the parental mice maintained on a standard rodent chow once they were colonized with bacteria and before being switched to their respective diets?
After colonizing the parental mice, we allowed the microbiomes to stabilize over the course of two weeks. During this time, mice were maintained on a standard chow that is rich in fiber (i.e. the FR diet in this study). After this two week period, the parental mice that were designated as FF were switched to the custom fiber-free chow. After a minimum 20 days, we set the breeding pairs. These details have been added to the section “Diet formulation and administration” of the Materials and Methods (lines 337-343).

b. Were the male breeders maintained on the experimental diets or the standard diet?
All parental mice were maintained on the same diet. This detail has been added to the section “Diet formulation and administration” of the Materials and Methods (lines 337-343).

c. When were the samples for parental microbiome composition analysis obtained and how were they collected?
The samples from parental mice were collected by fecal sampling 7 days post-oral gavage. This detail is specified in section “Bacterial culturing and colonization of 13- or 14-member synthetic microbiota (SM)” of the Materials and Methods: “Bacterial colonization among the parental mice was verified by qPCR of fecal DNA at 7 days post-oral gavage using strain-specific primers.” (lines 353-354).

d. How old were the pups when they were weaned?
The pups were weaned at 3 weeks of age. This detail has been added to the Materials and Methods (lines 332-333) and the study design schematic (**Fig 1A**).

e. How was the stream graph generated?
The stream plots were generated by pooling the abundances at each time point prior to plotting. This detail has been added to the figure legend “Abundances represent an average of all pups at the specified age” (lines 715-716) and to the Materials and Methods “*Streamplots based on the average abundance at each time point were created in Microsoft Excel 2016*” (lines 404-405).

6. Text clarifications:
a. Title: The manuscript does not test how immune system responds in the pups. Perhaps a more appropriate title would be to change "immune responses" to "immune cell populations," "mucosal immunity," or "colonic immunity."
The suggestion is well taken. We agree that immune responses may be slightly off base and have changed the title to "immune development" as we are examining changes in both immune cell populations and also transcriptomic differences by colonization and diet during early life development. We feel that this term accurately describes the scope of the immune characterizations undertaken while avoiding the inaccurate use of the term of "response". However, in case the reviewer still prefers any of the three suggested terms, we are open to accept it.

b. Line 27: Mice were fed fiber-free or fiber-rich diets.
Thanks for this comment; we have edited the abstract accordingly and now specify both of the diets that were used.

c. Lines 37, 191, 626: I do not think the data can distinguish whether there are differences in immune priming or in recruitment of immune cell subsets. Perhaps the phrasing "early colonic immune cell populations" would be more appropriate?
We agree with the point raised by the reviewer that the term "immune priming" is not be the most appropriate word choice. Here and elsewhere, we have instead used the term "immune development" to refer to the longitudinal cytokine expression profile or “immune profile” to refer to differences in immune cell populations according to colonization or diet.

d. Lines 71-73: The current phrasing implies that maternal diet primarily impacts the host epithelial transcriptome and that the presence or absence of *A. muciniphila* primarily impacts immune cell populations. However, the authors present data showing that both diet and *A. muciniphila* impact both host epithelial transcriptome and immune cells.
Indeed, it is true that the both diet and *A. muciniphila* impact different aspects/features of both the transcriptome and immune cells. We make the observation that maternal diet appears to have a more apparent/detectable impact on the host epithelial transcriptome on a gene set/pathway level, yet it is true that a greater number of differentially enriched transcripts were found in the comparison with or without the presence of *A. muciniphila*. We have rephrased the text as “*We show that both the maternal diet and the presence or absence of* A. muciniphila *are key contributing factors in shaping the host colonic transcriptome and the immune cell populations during this critical early life window*.” (Lines 71-73).

e. Line 86: Based on Tables S2 and S3, R. intestinalis and E. rectale are present in adult mice, but absence from pups.
We confirm that these taxa were not well transferred vertically, as mentioned in the Materials and Methods section "Bacterial culturing and colonization of 13- or 14-member synthetic microbiota (SM)". It is possible that these would be acquired later in life through coprophagy or that the starting colonization in parental mice was too low to be detectable in pups. We also note that the proliferation of these two fiber-degrading taxa depends on the chow used (e.g. better growth in adult mice fed the LabDiet # 5013), ostensibly due to the presence of specific glycan linkages that are not in the standard chow that we employed in this study. Of course, this in itself is a relevant consideration for replication in other labs, however the effects of variations in standard chows on the microbiome was not investigated here as only one FR diet was employed.

f. Line 88: Did these bacterial populations significantly expand due to increases in their absolute abundance or decreases in the absolute abundance of other members of the microbiome?
The data presented for these bacterial populations corresponds to that shown in Figure 1C, i.e. the relative abundances. While we adamantly agree that an assessment of absolute abundances would be important to consider, in practice, the amount of colon contents that can be isolated, especially at days 10 and 15, make such an approach challenging. This detail is discussed above.

g. Line 96: Please clarify if total RNA sequencing was performed on the total, proximal, or distal colon.
The RNA-Seq was performed on RNA isolated from the distal colon tissue. This had been clarified in the text under the section "Colonic transcriptome reflects differences in maternal diet" (lines 114-116): “*we performed total RNA sequencing on the distal colon tissues of 15*

day-old pups born to FR and FF 14SM dams as well as FR 13SM dams without A. muciniphila ***(Fig 2)***".

h. Line 128: The subtitle of this section is awkward.
We appreciate the feedback and have changed the subtitle to "Maternal fiber deprivation shapes microbiota-mediated immune development".

i. Lines 142-155: This paragraph is not very clear. If the manuscript needs to be shortened, this paragraph could be cut. If the authors would like to keep this paragraph, please clarify to which metabolites the authors are referring (line 145), reference figures 2D and 3A at line 149, and rework the sentence in lines 150-153.
We have opted to re-work the paragraph as we find the observations to be highly relevant to the overall study. In particular, we specify the AhR ligands in question (e.g. indoles derived from tryptophan), add the appropriate figure references, and have revised the text in lines 187-202 to make our point more clear:
"*Bacteria-derived metabolites represent a mechanistic link between changes in the microbiome and host immunity. While it would highly interesting to perform metabolomics analysis during this early life period, we note that the overall volume of colonic contents available is a major limitation, especially at 10-15 days of age. In the absence of such data, we would speculate that microbial metabolism of tryptophan, which is abundant in milk, may drive many of the observed microbiota-mediated immune changes. Microbial or host degradation of dietary tryptophan yields indole derivatives which can function as AhR ligands and stimulate production of IL-22 from ILC3s (Qiu et al, 2012; Li et al, 2018), both of which were elevated among pups of 14SM FF-fed dams at 15 days of age (****Fig 2D, 3A****). The expansion of these innate subsets also leads to apoptosis of commensal-specific CD4+ and CD8+ T cells (Hepworth et al, 2015; Liu et al, 2019). Likewise, among adaptive cell subsets, Th17 cells were significantly elevated among 14SM FR mice, potentially reflecting the effects of increased colonization with* A. muciniphila *in early life, since this bacterium can act as a direct AhR ligand via its outer membrane protein Amuc_1100 (Gu et al, 2021). While the absolute counts of regulatory T cells are expected to be lower in GF mice compared to colonized mice (Macpherson & Harris, 2004; Hrncir et al, 2008), in this study, a fixed number of cells were stained per organ, therefore we suspect that the higher proportions of adaptive cell subsets (namely CD8+, CD4+, Tregs, and Th1 cells) observed in GF mice may simply reflect a lower absolute number of innate cells (Macpherson & Harris, 2004; Kennedy et al, 2018).*"

j. Lines 160-162: The "lower induction" and "lower repression" is a little confusing on a quick first pass. If the authors are able to rephrase the comparison to make it a little clearer it may be helpful.
We agree that the sentence was worded in a confusing manner, especially as it contained multiple comparisons. We have revised the text to make these points more clearly:
"*Interestingly, an increase of NCR- ILC3 and Th17, and the decrease of CD8+ T cells, CD4+ T cells, Tregs and Th1 cells in pups born to 14SM FR-fed dams, relative to those of GF FR-fed dams, was not observed in pups born to 13SM dams, confirming a specific impact of* A. muciniphila *on these immune cell populations*." (lines 214-217).

k. Line 173: Please include "colonic" when describing the transcriptomic profile.
We specified the colon as the transcript source throughout the manuscript for clarity.

l. Line 195: Please add a "to" between "components" and "improve".
Thank you for catching this error. We have made the correction.

m. Line 577: Please add "targets" before "present once per genome."
We have revised this text as follows: "Distal colon contents were analyzed by qPCR using primers against targets that are unique to each bacteria and present once per genome."

(Line 715).

7. Refences to include:
a. Line 64: The claim that *A. muciniphila* is capable of degrading milk oligosaccharides should also reference Luna et al (2022) (doi: 10.1128/AEM.01487-21).
Thank you for the suggestion. We have included a reference to the article by Luna et al., as indicated (line 64, 279).

b. Line 64: The claim that *A. muciniphila* is an early colonizer of the gut is supported by Collado et al (2007) (DOI: 10.1128/AEM.01477-07).
Again, thanks for this suggestion. We have included a reference to Collado et al., in support of the claim that *Akkermansia* is an early colonizer of the gut (line 65).

c. Reference list: Some of the citations in the reference list do not show the full list of authors.
We have used the reference style for EMBO journals in Mendeley and also note that the journal requests "where there are more than 10 authors on a paper, 10 will be listed, followed by 'et al.'". In case the reviewer still finds error, we invite the reviewer to specify any references that are still missing authors—it is always possible there a mistake was made in importing reference metadata from existing repositories and we would endeavor to correct any such errors prior to publication.

d. The following articles may be important to mention when describing our current knowledge of whether maternal diet influences offspring immunity: Babu et al, (2018) (DOI: 10.1172/jci.insight.99223) and Thorburn et al, (2015) (doi: 10.1038/ncomms8320).
We have integrated these references into our discussion of the study findings, where appropriate (lines 180-182, 254-258). The Thorburn paper dovetails nicely with our suggestion of what disease models to investigate in follow-up works, whereas the Babu paper is very interesting as a complement to the observation of increased ILC3s in FF-fed mice since this aligns with their observation in a similar experimental setup. Despite being labelled a high-fat or fiber-free diet, the chows used in the two studies yield similar results. We would predict that microbial metabolic changes owing to a lack of fiber may be driving these shifts, however it would be prudent to isolate specific mechanisms in follow-up works.

8. Miscellaneous:
The authors' suggestion that differences in IgG in breast milk may explain the differences in *A. muciniphila* abundance in pups (Line 198) is an intriguing idea, especially since mice can generate anti-*Akkermansia* IgG1 antibodies (doi: 10.1126/science.aaw7479).
We greatly appreciate the reviewer's feedback on this suggestion. The Ansaldo 2019 paper referenced by the reviewer is indeed cited in manuscript (line 112), however we have added an explanation and brief connection to this paper again in the section where we suggest a link between *A. muciniphila* and IgG (line 267-270).

10th May 2023

Dear Prof. Desai,

Thank you for the submission of your revised manuscript to EMBO Molecular Medicine. I am pleased to inform you that we will be able to accept your manuscript pending the following final amendments:

1) Please address the referee #2 minor points.
2) Please check your manuscript carefully for grammar and syntax.
3) Please submit the manuscript as a "Research Article".
4) Synopsis: Please check your synopsis text and image before submission with your revised manuscript. Please be aware that in the proof stage minor corrections only are allowed (e.g., typos).
5) As part of the EMBO Publications transparent editorial process initiative (see our Editorial at http://embomolmed.embopress.org/content/2/9/329), EMBO Molecular Medicine will publish online a Review Process File (RPF) to accompany accepted manuscripts. This file will be published in conjunction with your paper and will include the anonymous referee reports, your point-by-point response and all pertinent correspondence relating to the manuscript. Let us know whether you agree with the publication of the RPF and as here, if you want to remove or not any figures from it prior to publication. Please note that the Authors checklist will be published at the end of the RPF.
6) Please provide a point-by-point letter INCLUDING my comments as well as the reviewer's reports and your detailed responses (as Word file).

I look forward to reading a new revised version of your manuscript as soon as possible.

* Please be reminded that your manuscript qualifies to have EMBO pay the open access publication. In order to qualify, it is important that you enter the following information when submitting your revised manuscript:

On the 'Financial Information' screen of the manuscript submission system, please select option #1: I (corresponding author) accept responsibility for paying the APC for this manuscript

There is nothing further that must be done at the revision submission stage.

When your paper is accepted for publication, you will be directed by the Wiley Author Services team to arrange payment. You should again indicate that you will pay via invoice, enter your details (and importantly, the VAT ID for your affiliated institute) and also include the following email address for receipt of the invoice: publishing@embo.org This will ensure that the invoice is directed to EMBO for payment

Yours sincerely,

Zeljko Durdevic

Zeljko Durdevic
Editor
EMBO Molecular Medicine

*** Instructions to submit your revised manuscript ***

*** PLEASE NOTE *** As part of the EMBO Publications transparent editorial process initiative (see our Editorial at https://www.embopress.org/doi/pdf/10.1002/emmm.201000094), EMBO Molecular Medicine will publish online a Review Process File to accompany accepted manuscripts.

In the event of acceptance, this file will be published in conjunction with your paper and will include the anonymous referee reports, your point-by-point response and all pertinent correspondence relating to the manuscript. If you do NOT want this file to be published, please inform the editorial office at contact@embomolmed.org.

When submitting your revised manuscript, please include:

1) a .docx formatted version of the manuscript text (including Figure legends and tables)

2) Separate figure files*

3) supplemental information as Expanded View and/or Appendix. Please carefully check the authors guidelines for formatting Expanded view and Appendix figures and tables at https://www.embopress.org/page/journal/17574684/authorguide#expandedview

4) a letter INCLUDING the reviewer's reports and your detailed responses to their comments (as Word file).

5) The paper explained: EMBO Molecular Medicine articles are accompanied by a summary of the articles to emphasize the major findings in the paper and their medical implications for the non-specialist reader. Please provide a draft summary of your article highlighting
- the medical issue you are addressing,
- the results obtained and
- their clinical impact.
This may be edited to ensure that readers understand the significance and context of the research.
Please refer to any of our published articles for an example.

6) For more information: There is space at the end of each article to list relevant web links for further consultation by our readers. Could you identify some relevant ones and provide such information as well? Some examples are patient associations, relevant databases, OMIM/proteins/genes links, author's websites, etc...

7) Author contributions: the contribution of every author must be detailed in a separate section.

8) EMBO Molecular Medicine now requires a complete author checklist (https://www.embopress.org/page/journal/17574684/authorguide) to be submitted with all revised manuscripts. Please use the checklist as guideline for the sort of information we need WITHIN the manuscript. The checklist should only be filled with page numbers were the information can be found. This is particularly important for animal reporting, antibody dilutions (missing) and exact values and n that should be indicted instead of a range.

9) Every published paper now includes a 'Synopsis' to further enhance discoverability. Synopses are displayed on the journal webpage and are freely accessible to all readers. They include a short stand first (maximum of 300 characters, including space) as well as 2-5 one sentence bullet points that summarise the paper. Please write the bullet points to summarise the key NEW findings. They should be designed to be complementary to the abstract - i.e. not repeat the same text. We encourage inclusion of key acronyms and quantitative information (maximum of 30 words / bullet point). Please use the passive voice. Please attach these in a separate file or send them by email, we will incorporate them accordingly.

You are also welcome to suggest a striking image or visual abstract to illustrate your article. If you do please provide a jpeg file 550 px-wide x 400-px high.

10) A Conflict of Interest statement should be provided in the main text

11) Please note that we now mandate that all corresponding authors list an ORCID digital identifier. This takes <90 seconds to complete. We encourage all authors to supply an ORCID identifier, which will be linked to their name for unambiguous name identification.

Currently, our records indicate that the ORCID for your account is 0000-0002-9223-2209.

Please click the link below to modify this ORCID:
Link Not Available

12) The system will prompt you to fill in your funding and payment information. This will allow Wiley to send you a quote for the article processing charge (APC) in case of acceptance. This quote takes into account any reduction or fee waivers that you may be eligible for. Authors do not need to pay any fees before their manuscript is accepted and transferred to our publisher.

*Additional important information regarding Figures

Each figure should be given in a separate file and should have the following resolution:
Graphs 800-1,200 DPI
Photos 400-800 DPI
Colour (only CMYK) 300-400 DPI"

Figures are not edited by the production team. All lettering should be the same size and style; figure panels should be indicated by capital letters (A, B, C etc). Gridlines are not allowed except for log plots. Figures should be numbered in the order of their appearance in the text with Arabic numerals. Each Figure must have a separate legend and a caption is needed for each panel.

*Additional important information regarding figures and illustrations can be found at https://bit.ly/EMBOPressFigurePreparationGuideline. See also figure legend preparation guidelines: https://www.embopress.org/page/journal/17574684/authorguide#figureformat

The system will prompt you to fill in your funding and payment information. This will allow Wiley to send you a quote for the article processing charge (APC) in case of acceptance. This quote takes into account any reduction or fee waivers that you may be eligible for. Authors do not need to pay any fees before their manuscript is accepted and transferred to our publisher.

***** Reviewer's comments *****

Referee #2 (Comments on Novelty/Model System for Author):

The authors have addressed most comments raised by the three reviewers and explained the limitations of the study. All three reviewers raised overlapping comments for which the authors offered detailed and careful responses. I now recommend the manuscript for publication at EMBO Journal.

Referee #2 (Remarks for Author):

The authors have addressed most comments raised by the three reviewers and explained the limitations of the study. All three reviewers raised overlapping comments for which the authors offered detailed and careful responses. I now recommend the manuscript for publication at EMBO Journal.

A few minor points should be addressed:

Editing:
In the new paragraph of the discussion on page 13 line 320 there is a typo, it should be the effects of the microbiome instead of the effects the microbiome. In the same line, it should read whereas instead of while.

Line 274 of page 11 it should be mouse pups instead of mice pups.

Line 196 page 8 should be bacterium-derived metabolites instead of bacteria-derived.

Discussion:
The citation of Lubin et al, 2023 was taken out of context. The paper shows how depriving ADULT mice from a diverse microbiota of adults by limiting the microbiome to the less diverse microbiome observed during the pre-weaning stages results in increase susceptibility to infection. Thus, the sentence should be edited and authors should simply acknowledge the limitations of a less diverse synthetic microbiota. Synthetic and diverse microbiota are indeed great tools to study mucosal immune responses. My question was simply related to which extent the 14SM microbiota recapitulates the intestinal immune responses seen in SPF mice.

***** Reviewer's comments *****

Referee #2 (Remarks for Author):

The authors have addressed most comments raised by the three reviewers and explained the limitations of the study. All three reviewers raised overlapping comments for which the authors offered detailed and careful responses. I now recommend the manuscript for publication at EMBO Journal.

A few minor points should be addressed:

Editing:
In the new paragraph of the discussion on page 13 line 320 there is a typo, it should be the effects of the microbiome instead of the effects the microbiome. In the same line, it should read whereas instead of while.
The typo and syntax errors have been corrected.
Line 274 of page 11 it should be mouse pups instead of mice pups.
This wording has been corrected.

Line 196 page 8 should be bacterium-derived metabolites instead of bacteria-derived.
We have used the singular form instead here, as suggested.

Discussion:
The citation of Lubin et al, 2023 was taken out of context. The paper shows how depriving ADULT mice from a diverse microbiota of adults by limiting the microbiome to the less diverse microbiome observed during the pre-weaning stages results in increase susceptibility to infection. Thus, the sentence should be edited and authors should simply acknowledge the limitations of a less diverse synthetic microbiota. Synthetic and diverse microbiota are indeed great tools to study mucosal immune responses. My question was simply related to which extent the 14SM microbiota recapitulates the intestinal immune responses seen in SPF mice.
Thank you for clarifying the scope of the initial question. We have addressed the question of how 14SM immune profiles compare to SPF mice in lines 294-301. Our discussion of the Lubin article is not directly in response to this question, rather, we bring it up to highlight the challenges and limitations of using a defined microbiota to study immune interactions in early life.

*****

16th May 2023

Dear Prof. Desai,

We are pleased to inform you that your manuscript is accepted for publication and is now being sent to our publisher to be included in the next available issue of EMBO Molecular Medicine.

Please read below for additional IMPORTANT information regarding your article, its publication and the production process.

Congratulations on your interesting work,

Zeljko Durdevic

Zeljko Durdevic
Editor
EMBO Molecular Medicine

Follow us on Twitter @EmboMolMed
Sign up for eTOCs at embopress.org/alertsfeeds

*** *** *** IMPORTANT INFORMATION *** *** ***

SPEED OF PUBLICATION
The journal aims for rapid publication of papers, using using the advance online publication "Early View" to expedite the process: A properly copy-edited and formatted version will be published as "Early View" after the proofs have been corrected. Please help the Editors and publisher avoid delays by providing e-mail address(es), telephone and fax numbers at which author(s) can be contacted.

Should you be planning a Press Release on your article, please get in contact with embomolmed@wiley.com as early as possible, in order to coordinate publication and release dates.

LICENSE AND PAYMENT:

All articles published in EMBO Molecular Medicine are fully open access: immediately and freely available to read, download and share.

EMBO Molecular Medicine charges an article processing charge (APC) to cover the publication costs. You, as the corresponding author for this manuscript, should have already received a quote with the article processing fee separately. Please let us know in case this quote has not been received.

Once your article is at Wiley for editorial production you will receive an email from Wiley's Author Services system, which will ask you to log in and will present you with the publication license form for completion. Within the same system the publication fee can be paid by credit card, an invoice, pro forma invoice or purchase order can be requested.

Payment of the publication charge and the signed Open Access Agreement form must be received before the article can be published online.

PROOFS

You will receive the proofs by e-mail approximately 2 weeks after all relevant files have been sent o our Production Office. Please return them within 48 hours and if there should be any problems, please contact the production office at embopressproduction@wiley.com.

Please inform us if there is likely to be any difficulty in reaching you at the above address at that time. Failure to meet our

deadlines may result in a delay of publication.

All further communications concerning your paper proofs should quote reference number EMM-2022-17241-V3 and be directed to the production office at embopressproduction@wiley.com.

Thank you,

Zeljko Durdevic
Editor
EMBO Molecular Medicine

EMBO Press Author Checklist

Corresponding Author Name: Mahesh S Desai	
Journal Submitted to: EMBO Molecular Medicine	
Manuscript Number: EMM-2022-17241	

USEFUL LINKS FOR COMPLETING THIS FORM
The EMBO Journal - Author Guidelines
EMBO Reports - Author Guidelines
Molecular Systems Biology - Author Guidelines
EMBO Molecular Medicine - Author Guidelines

Reporting Checklist for Life Science Articles (updated January 2022)
This checklist is adapted from Materials Design Analysis Reporting (MDAR) Checklist for Authors. MDAR establishes a minimum set of requirements in transparent reporting in the life sciences (see Statement of Task: 10.31222/osf.io/9sm4x). Please follow the journal's guidelines in preparing your manuscript.
Please note that a copy of this checklist will be published alongside your article.

Abridged guidelines for figures
1. Data
The data shown in figures should satisfy the following conditions:
- the data were obtained and processed according to the field's best practice and are presented to reflect the results of the experiments in an accurate and unbiased manner.
- ideally, figure panels should include only measurements that are directly comparable to each other and obtained with the same assay.
- plots include clearly labeled error bars for independent experiments and sample sizes. Unless justified, error bars should not be shown for technical replicates.
- if n<5, the individual data points from each experiment should be plotted. Any statistical test employed should be justified.
- Source Data should be included to report the data underlying figures according to the guidelines set out in the authorship guidelines on Data Presentation.

2. Captions
Each figure caption should contain the following information, for each panel where they are relevant:
- a specification of the experimental system investigated (eg cell line, species name).
- the assay(s) and method(s) used to carry out the reported observations and measurements.
- an explicit mention of the biological and chemical entity(ies) that are being measured.
- an explicit mention of the biological and chemical entity(ies) that are altered/varied/perturbed in a controlled manner.
- the exact sample size (n) for each experimental group/condition, given as a number, not a range;
- a description of the sample collection allowing the reader to understand whether the samples represent technical or biological replicates (including how many animals, litters, cultures, etc.).
- a statement of how many times the experiment shown was independently replicated in the laboratory.
- definitions of statistical methods and measures:
  - common tests, such as t-test (please specify whether paired vs. unpaired), simple χ2 tests, Wilcoxon and Mann-Whitney tests, can be unambiguously identified by name only, but more complex techniques should be described in the methods section;
  - are tests one-sided or two-sided?
  - are there adjustments for multiple comparisons?
  - exact statistical test results, e.g., P values = x but not P values < x;
  - definition of 'center values' as median or average;
  - definition of error bars as s.d. or s.e.m.

Please complete ALL of the questions below.
Select "Not Applicable" only when the requested information is not relevant for your study.

Materials

Newly Created Materials	Information included in the manuscript?	In which section is the information available? (Reagents and Tools Table, Materials and Methods, Figures, Data Availability Section)
New materials and reagents need to be available; do any restrictions apply?	Not Applicable	

Antibodies	Information included in the manuscript?	In which section is the information available? (Reagents and Tools Table, Materials and Methods, Figures, Data Availability Section)
For **antibodies** provide the following information:<br>- Commercial antibodies: RRID (if possible) or supplier name, catalogue number and or/clone number<br>- Non-commercial: RRID or citation	Yes	Materials and Methods

DNA and RNA sequences	Information included in the manuscript?	In which section is the information available? (Reagents and Tools Table, Materials and Methods, Figures, Data Availability Section)
**Short novel DNA or RNA including primers, probes**: provide the sequences.	Yes	Supplementary Tables

Cell materials	Information included in the manuscript?	In which section is the information available? (Reagents and Tools Table, Materials and Methods, Figures, Data Availability Section)
**Cell lines:** Provide species information, strain. Provide accession number in repository **OR** supplier name, catalog number, clone number, and/**OR** RRID.	Not Applicable	
**Primary cultures:** Provide species, strain, sex of origin, genetic modification status.	Not Applicable	
Report if the cell lines were recently **authenticated** (e.g., by STR profiling) and tested for mycoplasma contamination.	Not Applicable	

Experimental animals	Information included in the manuscript?	In which section is the information available? (Reagents and Tools Table, Materials and Methods, Figures, Data Availability Section)
**Laboratory animals or Model organisms:** Provide species, strain, sex, age, genetic modification status. Provide accession number in repository **OR** supplier name, catalog number, clone number, **OR** RRID.	Yes	Materials and Methods
**Animal observed in or captured from the field:** Provide species, sex, and age where possible.	Not Applicable	
Please detail **housing and husbandry conditions**.	Yes	Materials and Methods

Plants and microbes	Information included in the manuscript?	In which section is the information available? (Reagents and Tools Table, Materials and Methods, Figures, Data Availability Section)
**Plants:** provide species and strain, ecotype and cultivar where relevant, unique accession number if available, and source (including location for collected wild specimens).	Not Applicable	
**Microbes:** provide species and strain, unique accession number if available, and source.	Yes	Materials and Methods

Human research participants	Information included in the manuscript?	In which section is the information available? (Reagents and Tools Table, Materials and Methods, Figures, Data Availability Section)
If collected and within the bounds of privacy constraints report on age, sex and gender or ethnicity for all study participants.	Not Applicable	

Core facilities	Information included in the manuscript?	In which section is the information available? (Reagents and Tools Table, Materials and Methods, Figures, Data Availability Section)
If your work benefited from core facilities, was their service mentioned in the acknowledgments section?	Yes	Materials and Methods

Design

Study protocol	Information included in the manuscript?	In which section is the information available? (Reagents and Tools Table, Materials and Methods, Figures, Data Availability Section)
If study protocol has been **pre-registered, provide DOI in the manuscript**. For clinical trials, provide the trial registration number **OR** cite DOI.	Not Applicable	
Report the **clinical trial registration number** (at ClinicalTrials.gov or equivalent), where applicable.	Not Applicable	

Laboratory protocol	Information included in the manuscript?	In which section is the information available? (Reagents and Tools Table, Materials and Methods, Figures, Data Availability Section)
Provide DOI OR other citation details if **external detailed step-by-step protocols** are available.	Yes	Materials and Methods

Experimental study design and statistics	Information included in the manuscript?	In which section is the information available? (Reagents and Tools Table, Materials and Methods, Figures, Data Availability Section)
Include a statement about **sample size** estimate even if no statistical methods were used.	Yes	Materials and Methods, Figures
Were any steps taken to minimize the effects of subjective bias when allocating animals/samples to treatment (e.g. **randomization procedure**)? If yes, have they been described?	Not Applicable	
Include a statement about **blinding** even if no blinding was done.	Yes	Materials and Methods
Describe **inclusion/exclusion criteria** if samples or animals were excluded from the analysis. Were the criteria pre-established?<br><br>If sample or data points were omitted from analysis, report if this was due to attrition or intentional exclusion and provide justification.	Yes	Materials and Methods
For every figure, are **statistical tests** justified as appropriate? Do the data meet the assumptions of the tests (e.g., normal distribution)? Describe any methods used to assess it. Is there an estimate of variation within each group of data? Is the variance similar between the groups that are being statistically compared?	Yes	Materials and Methods, Figures

Sample definition and in-laboratory replication	Information included in the manuscript?	In which section is the information available? (Reagents and Tools Table, Materials and Methods, Figures, Data Availability Section)
In the figure legends: state number of times the experiment was **replicated** in laboratory.	Yes	Materials and Methods
In the figure legends: define whether data describe **technical or biological replicates**.	Yes	Materials and Methods

Ethics

Ethics	Information included in the manuscript?	In which section is the information available? (Reagents and Tools Table, Materials and Methods, Figures, Data Availability Section)
Studies involving **human participants**: State details of **authority granting ethics approval** (IRB or equivalent committee(s), provide reference number for approval.	Not Applicable	
Studies involving **human participants**: Include a statement confirming that **informed consent** was obtained from all subjects and that the experiments conformed to the principles set out in the WMA Declaration of Helsinki and the Department of Health and Human Services Belmont Report.	Not Applicable	
Studies involving **human participants:** For publication of **patient photos**, include a statement confirming that consent to publish was obtained.	Not Applicable	
Studies involving experimental **animals**: State details of **authority granting ethics approval** (IRB or equivalent committee(s), provide reference number for approval. Include a statement of compliance with ethical regulations.	Yes	Materials and Methods
Studies involving **specimen and field samples:** State if relevant **permits** obtained, provide details of authority approving study; if none were required, explain why.	Not Applicable	

Dual Use Research of Concern (DURC)	Information included in the manuscript?	In which section is the information available? (Reagents and Tools Table, Materials and Methods, Figures, Data Availability Section)
Could your study fall under dual use research restrictions? Please check biosecurity documents and list of **select agents and toxins** (CDC): https://www.selectagents.gov/sat/list.htm	Not Applicable	
If you used a select agent, is the security level of the lab appropriate and reported in the manuscript?	Not Applicable	
If a study is subject to dual use research of concern regulations, is the name of the **authority granting approval and reference number** for the regulatory approval provided in the manuscript?	Not Applicable	

Reporting

The MDAR framework recommends adoption of discipline-specific guidelines, established and endorsed through community initiatives. Journals have their own policy about requiring specific guidelines and recommendations to complement MDAR.

Adherence to community standards	Information included in the manuscript?	In which section is the information available? (Reagents and Tools Table, Materials and Methods, Figures, Data Availability Section)
State if relevant guidelines or checklists (e.g., **ICMJE, MIBBI, ARRIVE, PRISMA**) have been followed or provided.	Not Applicable	
For **tumor marker prognostic studies,** we recommend that you follow the **REMARK** reporting guidelines (see link list at top right). See author guidelines, under 'Reporting Guidelines'. Please confirm you have followed these guidelines.	Not Applicable	
For **phase II and III randomized controlled trials**, please refer to the **CONSORT** flow diagram (see link list at top right) and submit the CONSORT checklist (see link list at top right) with your submission. See author guidelines, under 'Reporting Guidelines'. Please confirm you have submitted this list.	Not Applicable	

Data Availability

Data availability	Information included in the manuscript?	In which section is the information available? (Reagents and Tools Table, Materials and Methods, Figures, Data Availability Section)
Have **primary datasets** been deposited according to the journal's guidelines (see 'Data Deposition' section) and the respective accession numbers provided in the Data Availability Section?	Yes	Materials and Methods
Were **human clinical and genomic datasets** deposited in a public access-controlled repository in accordance to ethical obligations to the patients and to the applicable consent agreement?	Not Applicable	
Are **computational models** that are central and integral to a study available without restrictions in a machine-readable form? Were the relevant accession numbers or links provided?	Not Applicable	
If publicly available data were reused, provide the respective **data citations in the reference list**.	Not Applicable	
